# Children use algorithm induction to discover patterns in data

**DOI:** 10.1038/s41467-026-73029-9

**Published:** 2026-05-30

**Authors:** Benjamin Pitt, Elena Leib, David O’Shaughnessy, Charlene Gallardo, Stephen Ferrigno, Steven T. Piantadosi

**Affiliations:** 1 Department of Psychology, University of California, Berkeley, Berkeley, CA USA; 2 Department of Social and Behavioral Sciences, Toulouse School of Economics, Toulouse, France; 3 School of Molecular and Life Sciences, Curtin University, Bentley, WA Australia; 4 Department of Psychology, University of Wisconsin, Madison, Madison, WI USA

**Keywords:** Human behaviour, Learning and memory

## Abstract

Humans are unique in our ability to acquire diverse skills and inhabit myriad environments, but the cognitive mechanisms underlying such fast, flexible learning remain unresolved. Inspired by theories of artificial intelligence, here we show evidence for one such learning mechanism - program induction - in US American and indigenous Tsimane’ children in the Bolivian Amazon. Participants viewed novel patterns and were asked to generalize them to new stimuli, alphabets, and lengths, without feedback. Given very limited data, participants across ages, cultures, and conditions constructed response patterns that shared abstract structure with the sample patterns. Computational modeling shows that responses likely reflect discovery of latent rules, rather than simple heuristics or associations, even among children without formal schooling. The results suggest program induction serves as a domain-general learning mechanism from early in life, allowing children across cultures to rapidly infer the algorithmic structure of their natural and cultural environment, whatever it might be.

## Abstract (web summary)

Humans are extraordinarily fast, flexible learners. Here, the authors show evidence that this capacity is powered by a general-purpose learning mechanism that allows children across cultures to discover patterns in data, even without instruction.

## Introduction

Humans are extraordinary learners. Many animals can innately perform elaborate behaviors like singing songs, building webs, or navigating by the stars^1–5^. In some species, individuals learn novel skills from their conspecifics, like how to crack nuts or fly efficient migratory paths^6–8^. But humans are outliers: Although born relatively helpless, children rapidly develop rich bodies of knowledge^9^, theories^10–12^, and skills^13^ that are unparalleled in other species. Long before their first day of school, children can speak and understand language, infer the hidden causes of events and intentions of other people, predict the outcomes of physical systems, learn the statistical regularities of their environment, and discover abstract relations between objects, among other cognitive feats^14^.

To explain the astonishing speed of human learning, some researchers have posited innate mechanisms specialized for learning language, object perception, causal inference, spatial relations, geometry, numerical perception, and other fundamental domains^15–24^. However, human learning extends beyond these basic domains of knowledge to a wide variety of skills, practices, and cognitive abilities with no clear biological basis. For example, in many industrialized cultures, children learn to tie their shoes, take the bus, and play soccer – skills that are culturally transmitted among individuals, not genetically inherited across generations^25^. Moreover, people develop dramatically different abilities depending on the specifics of their cultural and ecological setting, from ice fishing to abacus calculation to planting and foraging^26–29^. Some of these skills are supported by explicit instruction^30^, but many depend primarily on individual experimentation, play, or observation^31–33^. This extraordinary cognitive flexibility has allowed our species to populate a wide variety of natural environments around the world and to innovate new technological, social, and political tools^34–39^ but the cognitive mechanisms that underlie this “improvisational intelligence”^25,40^ remain unresolved: How do children learn so much, so flexibly? The enormous diversity of human abilities suggests we may have a very general cognitive mechanism for learning the structure of our environment.

One proposal for such a general learning mechanism comes from theories of artificial intelligence^41,42^. On this account, learning is driven by *program induction*, a process by which intelligent agents observe data—whether a string of sounds, an array of shapes, or any other sensory input—and infer the computational processes that likely generated it, like scientists developing formalized theories to explain their data^43^. These algorithms can be formalized in a “Language of Thought”^44–46^ a collection of primitive functions that can compose larger functions, much like operations in a computer program. These languages need not be large or tailored to any particular domain – rather, even very small languages of thought (i.e., those composed of few primitive operations) can be universal in their expressive power, capable of representing all possible computations (e.g., ref. ^47^).

Such inductive theories have been used to explain human learning as a process of constructing concise mental programs across a wide variety of populations and domains, including language^48^, physical intuitions^49^, social reasoning^50^, numerical development^51^, and geometric pattern-learning^20,52–55^, among many others^9,13,46,52,56–65^. However, people’s success in these domains could, in principle, reflect domain-specific abilities that are either learned socially by individuals or inherited genetically across generations of learners. Evaluating the role of domain-general learning mechanisms requires testing naive learners in domains that are not plausible targets of natural selection or cultural transmission.

To that end, some researchers have tested the ability of infants and young children to learn the abstract structure of novel patterns, which can reflect any number of algorithms with arbitrary semantic structure. For example, studies of seven-month old infants show that they can learn the rules governing short sequences of images or spoken sounds long before they have learned to talk, distinguishing familiar patterns from unfamiliar patterns, even when the specific stimuli have all changed (e.g., AAB vs. ABB;^66–68^; also see refs. ^69,70^). These early pattern-learning abilities are found in more open-ended tasks as well: When presented with novel sequences of colored shapes (e.g., ), preschool-aged children across cultures can identify the repeating unit (), insert missing elements, extend the patterns to new lengths, and translate them to new stimuli, with variable success^71–77^. While some of these patterning tasks can be solved using simple heuristics like one-to-one duplication or translation, others require reasoning about the abstract relations among items^78–80^, an ability believed to be fundamental to cognitive development across domains^66,81–83^. Indeed, children’s success in these pattern-learning tasks correlates with domain-general cognitive abilities like relational knowledge, cognitive flexibility, and inhibitory control, and predicts performance in reading, numeracy, mathematical reasoning, and problem-solving tasks, even controlling for other factors^71,72,75,84–87^. These findings from infants and children suggest that patterning ability is somehow central to cognitive development, but the role it plays and the learning mechanisms that underlie it remain unclear^70,80,84,87,88^. Inspired by this literature, we combined patterning tasks with novel computational techniques to clarify whether and how children across cultures abstract arbitrary algorithmic structure from small amounts of data.

One-hundred forty one children (ages 3–13 years) from two cultures viewed novel patterns of colors and/or shapes and then were asked to generalize them to new lengths and new stimuli, without instruction or feedback. In Experiment 1, we presented participants with short action sequences (i.e., sorting colored balls into bins; e.g., -left, -right, -left, -right) and asked them to continue each sequence. In Experiment 2, we presented children with static arrays of geometric shapes (e.g., ) and asked them to make similar patterns, but critically using different lengths and stimuli. Both the tasks and the patterns themselves were likely unfamiliar to participants, requiring them to discover arbitrary structure in a novel domain without specific cultural or biological support.

**Fig. 1:**
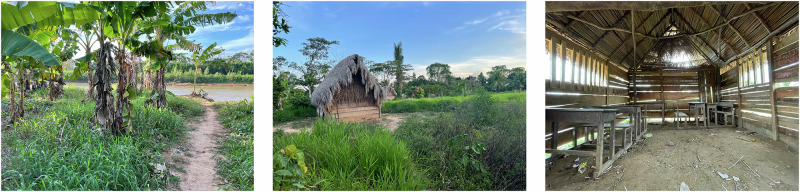
The Tsimane’ context. Tsimane' villages are situated along the Maniqui river (left) in the Bolivian Amazon, and are typically composed of simple homes (center) and a school house (right).

We focused our experimental work on a population which provides an especially stringent test of generalizability across cultures: indigenous Tsimane’ children in lowland Bolivia. The Tsimane’ are a small-scale society of the Amazon basin, where they rely primarily on hunting, gathering, fishing, and farming for subsistence (see Fig. 1;^89,90^). Although formal schooling is now available in many villages, the regularity and quality of this schooling is highly variable, as in other indigenous groups^91^. As a consequence, many Tsimane’ adults have little or no formal schooling and do not read, write, or use math^91–93^. Tsimane’ children spend much of their time outside, exploring large swaths of the forest on foot with other children^94^. They acquire a wide variety of skills before reproductive age, including in food production (e.g., fishing, hunting, gardening), building and crafts (e.g., making houses, textiles, and canoes), and childcare (the average Tsimane’ has about 8 siblings;^27,95–97^), as well as rich ethnobotanical knowledge^98,99^. In many ways, the experience of Tsimane’ children differs dramatically from that of urban US American children, including the ecological setting, subsistence style, social scale, level of industrialization, language, and material culture. Although no one of these differences was of specific interest, these distinct populations allowed us to test whether participants’ inductive abilities were robust to large cultural differences.

In addition to culture, age, and formal schooling, a primary individual variable that we used was children’s knowledge of counting, an algorithm found across human cultures that develops relatively early in life. Number is also a cognitive domain for which profoundly different developmental theories have been articulated, from strongly nativist accounts positing pre-verbal counting mechanisms^24,100–102^, to strongly empiricist accounts positing that numerical abilities are learned^19,88,103^, including via procedural learning^104,105^ and algorithm induction^51,106^. A variety of developmental and computational studies have charted the acquisition of counting abilities^19,24,104,107–110^ and show that children progress through discrete stages of number knowledge^19,111–113^. Children who have mastered the logic of counting are called *Full-counters*; Our analyses focus on those who have not yet reached this inflection point in numerical cognition, so-called *Subset-knowers*.

If program induction drives the acquisition of algorithms like the counting algorithm, then even subset-knowers and unschooled children should be capable of program induction. Alternatively, program induction could be available to only some children or used in only some tasks. For example, without sufficient schooling, instruction, or cultural support, children’s patterning ability could be limited to simple imitation^114^ or association (e.g., transition probabilities;^115–118^). Likewise, children might be unable to apply any latent program induction capacities to our tasks if, for example, the specific stimuli we chose were not clearly distinguishable to them. In such cases, participants might be able to replicate some aspects of the target pattern, but unable to perform the kind of abstraction and generalization that characterizes cognition in many domains among human adults.

Here, we characterized the inferential processes underlying children’s skill learning by analyzing participants’ response patterns in two primary ways. First, we used measures of accuracy and edit distance that quantify how well participants incorporated into their responses various features of the specific sample patterns they saw. Second, we used a Language of Thought model to infer the mental program that participants likely used to generate each response pattern. This approach allowed us to characterize not just *whether* participants understood the sample patterns, but also *what* they learned (i.e., which logical features) and *how* they learned it (i.e., by inducing implicit algorithmic structure or tracking simple statistical regularities).

## Results

### Experiment 1: Extending sequences of actions

**Fig. 2:**
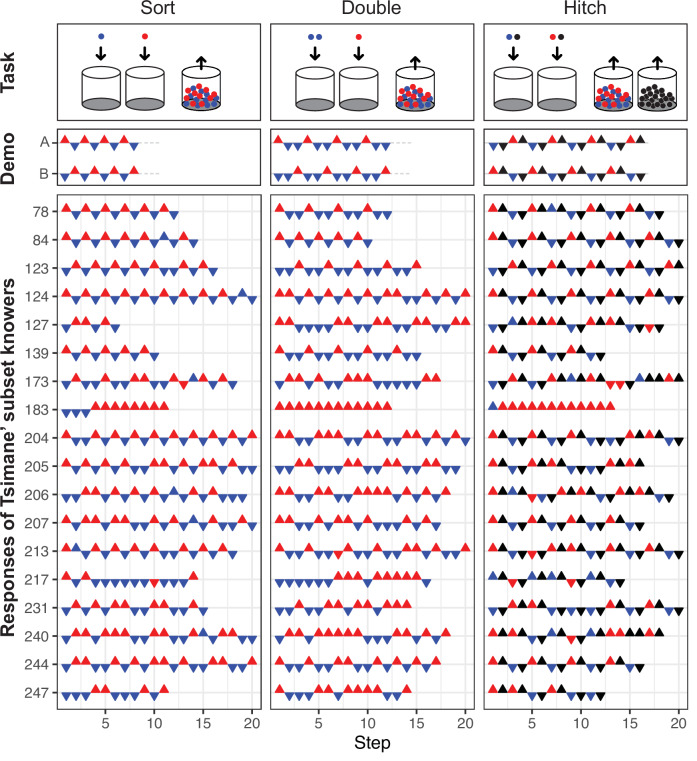
Action sequences in Experiment 1. Participants performed three ball-sorting tasks. Top: In demonstrating each task, the experimenter moved colored balls from one or more source bins into two or more response bins according to a predefined algorithm, as shown above each set of response bins. Bottom: After an experimenter demonstrated four steps, participants were asked to continue the procedure without feedback. Each row shows the response patterns of a Tsimane' subset-knower: Each triangle represents a ball; The color of the triangle reflects the color of the ball; Upward triangles show balls placed in the left bin; Downward triangles show balls placed in the right bin.

In the first experiment, 97 children from two cultures – 24 US Americans and 72 Tsimane’ – observed an experimenter move colored balls into bins and then continued the sequence, without instruction or feedback (see *Methods*). As shown at the top of Fig. 2, we used three sample patterns, which varied in complexity. In SORT, we sorted balls by color into two side-by-side bins, alternating after each ball (e.g., -left, -right, -left, -right...).

In DOUBLE, we alternated both color and number (e.g., -left, -right...). In HITCH, we sorted two colors while including a third color at each step (-left, -right). In their responses, participants were limited only in time to approximately 50 seconds per response pattern. In addition to these algorithm-induction tasks, participants completed standard tests of their basic number knowledge, including the Give-N task to classify them as Full-counters or Subset-knowers (see *Methods*).

Overall, participants used 4 to 32 balls in their responses (mean = 20  ± 5.45). Figure 2 (bottom) visualizes the responses from a representative sample of Tsimane’ Subset-knowers in detail. The colors of the triangles reflect the colors of the balls the child placed in a response bin, as well as which bin (upward is the left bin, downward is the right), and the ordering reflects the sequence of their actions (arranged from left to right). If participants were unable to learn the observed pattern, they might have produced responses that were largely random, responses that corresponded to a single pattern, or no responses at all. Instead, inspection of the responses reveals systematic patterns with respect to color, number, and bin, even in these subset-knowers. For example, children in the SORT task typically understood that the two colors should go into two different bins (up vs. down triangles) and most children understood that they should alternate between reds and blues (e.g., Participants 123, 124). Even in cases where they did not induce the intended algorithm, children appear to follow closely-related algorithms, and rarely appear to respond randomly. For example, participant 183 appears to have understood that colors should go in separate bins, but sorted by first choosing a sequence of blues and then choosing a sequence of reds. Participant 240 appears to follow some alternation, but not one-to-one. Similarly, children in DOUBLE and HITCH generally provided systematic responses that captured at least some, if not all, aspects of the sample patterns. For example, in HITCH, participant 173 correctly selected a black ball every other time while alternating colors, but did not always place reds and blues in different bins. Others performed this task as expected (e.g., participants 84, 123, and 124).

**Fig. 3:**
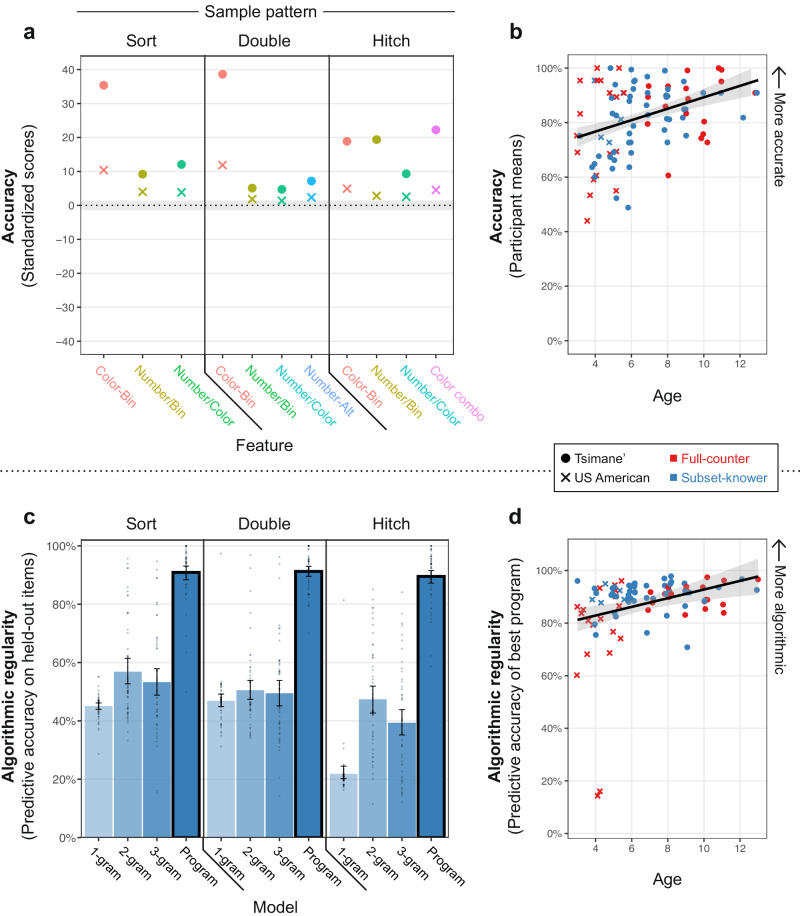
Results of Experiment 1 in subset-knowers only (*N* = 57). **a** Feature-wise analysis of responses. Colored points show standardized performance on six measures of accuracy, such that a point at *Y *= 15 is 15 standard deviations from the chance mean (dotted line). The gray region shows 95% confidence interval around chance. **b** Each point shows a participant’s mean accuracy across measures, and gray bands show 95% confidence intervals around the regression line. Accuracy improved with age, with no reliable difference between Subset-knowers and Full-counters. **c** Results of generative Bayesian modeling. Bolded bars show how often, on average, the most likely program correctly predicted the (masked) elements of each response, points show individual participant values, and error bars show bootstrapped 95% confidence intervals. Lighter bars show the same measure for n-gram models, whose predictions depend only on local context. **d** Each point shows a participant’s mean algorithmic regularity, and gray bands show 95% confidence intervals around the regression line. Older children tended to produce more algorithmic responses, with no statistically significant difference between Subset-knowers and Full-counters.

To quantify accuracy, we measured how well responses captured various features of each sample pattern, including the assignment of colors to bins, the number of balls per step, the alternation of color, and the alternation of number (see *Methods*). This approach treats each response as potentially noisy so that small errors (e.g., a single addition, deletion, or substitution) do not disqualify otherwise correct responding. To compare performance to chance, we permuted participants’ responses (within participant and task) and recomputed our eleven accuracy measures 10,000 times. The resulting chance distribution defines the Y-axis in Fig. 3a, where the horizontal gray bar shows the 95% confidence interval around chance performance.

As that figure shows, Tsimane’ subset-knowers were reliably above chance on every measure for every pattern (*ps* < .001) by 4–38 standard deviations. Our sample of US subset-knowers also performed above chance on every measure in every pattern (*ps* < .010; see *Supplemental Information* for detailed results), except in Double, where their scores on Number/Color and Number/Bin did not differ reliably from chance. Groups did not differ significantly in their accuracy (*t*(83.24) = − 0.07, *p* = . 948, *β* = − . 004, 95%CI[-.13, .12], *N* = 84) however US children generally had lower accuracy scores than Tsimane’ children, a trend that may reflect cross-group differences in age: the children in our US sample were on average younger than those in our Tsimane’ sample (since subset-knowers can be found at older ages among Tsimane’). Collapsing across measures and groups, participants’ accuracy was high at all ages (mean = 82%, SEM = 1%), and was positively correlated with age (even controlling for group, education, and number knowledge; *t*(82.86) = 2.36, *p* = .021, *β* = . 06, 95%CI[.01, .11], *N *= 97), with no statistically significant difference between subset-knowers and full-counters (*t*(82.42) = − 1.087, *p* = . 280, *β* = . 06, 95%CI[-.05, .16] *N *= 97; see Fig. 3b). Accuracy increased with schooling (*t*(82.60) = 3.190, *p* = . 002, *β* = . 03, 95%CI[.01, .06], *N* = 84), but this effect was not significant when controlling for age and other covariates (*t*(82.41) = 0.01, *p* = . 995, *β* = . 001, 95%CI[− .04, .04], *N* = 84).

This pattern of results suggests that children across cultures and ages can infer the structure of novel patterns even before they have learned to count, and that this understanding does not reflect a single moment of insight, but rather a piecemeal process of inferring the individual algorithmic features of the input. Such stepwise processes are consistent with constructivist theories of cognitive development^11^ and characterize learning in a variety of cognitive domains: For example, when learning to talk, draw, or count, children start by making incomplete (and sometimes erroneous) inferences about the grammar of their native language^119^, the salient features of common objects^120^, or the central rules of exact quantification^103,121,122^. Likewise here, the inductive process was not all-or-nothing: children often inferred some of the features of the sample patterns, while missing others.

Next, we used a computational model to quantify the amount of algorithmic structure present in each response pattern - that is, how well did it follow a rule. To do this, we masked each token (i.e., ball and container pair) in each response sequence, and attempted to predict the masked tokens from a Bayesian program-induction model that was trained on the rest of each individual’s response sequence. If the sequence contained a pattern, then a masked token should be predictable from the others. Importantly, if children’s responses contained interesting algorithmic structure, then a model that inferred algorithms should make better predictions than a model that only tracks token frequencies or transitional probabilities. Candidate programs were composed by combining a small set of domain-general primitives in a Language of Thought (like LENGTH, REPEAT, and OR; see *Methods* for full set)^13,44,46,52,61,62,123,124^). The most likely programs were those that provided the best balance between concision (i.e., a simple program) and predictive accuracy on the masked items (i.e., a good description of the observed data; see *Methods*). This approach allowed us to objectively quantify which programs—if any—participants were likely using to generate their responses, even when those responses deviated unpredictably from the sample pattern.

As shown in Fig. 3c, the best programs correctly predicted the missing token in subset-knowers’ responses 90% of the time on average, indicating highly algorithmic responding. As shown in Fig. 3d, responses were highly algorithmic at all ages and knower-levels, with no statistically significant difference between full-counters and subset-knowers (*t*(245) = 1.25, *p* = .214, *β* = .02, 95%CI[− .01, .06], *N *= 97). Algorithmic regularity tended to increase with age, although this trend was not significant when controlling for education, group, and numerical knower-level (*t*(245) = 1.18, *p* = .241, *β* = . 01, 95%*CI*[ − . 001, . 04], *N* = 97). Formal schooling likewise had no statistically significant effect on algorithmic regularity (*t*(245) = − 0.05, *p* = . 962, *β* = − . 0004, 95%*CI*[ − 0.02, .02], *N *= 97), even when it was the sole predictor of performance (*t*(251) = 0.75, *p* = .454, *β* = .004, 95%*CI*[ − 0.01, . 01], *N *= 97; see Supplementary Fig. S2 in *Supplemental Information*). Tsimane’ participants scored higher on average than US Americans (*t*(245) = 2.25, *p* = .025, *β* = . 06, 95%*CI*[. 01, . 12], *N *= 97), controlling for other factors. Importantly, we compared the performance of this program-induction model to simpler models that only represent the token frequencies (unigram), pairs of tokens (bigram), or triplets of tokens (trigram) in the response pattern. If participants were responding randomly, then their response patterns should be well-described by a unigram model, which is sensitive only to the base rate of each token (i.e., block type). If participants were simply learning associations among block types, as if “chunking” the patterns into units of two or three actions^125^, then their response patterns should be well-described by bigram and trigram models. On the contrary, the program-induction model outperformed all of these alternatives in all sub-groups: Among Tsimane’ and American children, in subset-knowers and full-counters, and even among the 21 children who had never attended school, exceeding the next best model on average by 35% and exceeding chance performance by 50%. Program models were the best predictor for 95 of our 97 participants.

These findings are consistent with the proposal that children across cultures infer the logical structure of novel patterns using program induction. However, in principle, participants could succeed in this task in part by memorizing and reproducing the same set of actions that they observed - imitating their form rather than inferring their abstract structure. To better characterize the learning mechanisms, we conducted a second experiment in which children were asked to not only reproduce novel patterns, but also to generalize them to new lengths and new stimuli, a task for which imitation and simple association learning are insufficient.

### Experiment 2: Generalizing patterns of shapes

**Fig. 4:**
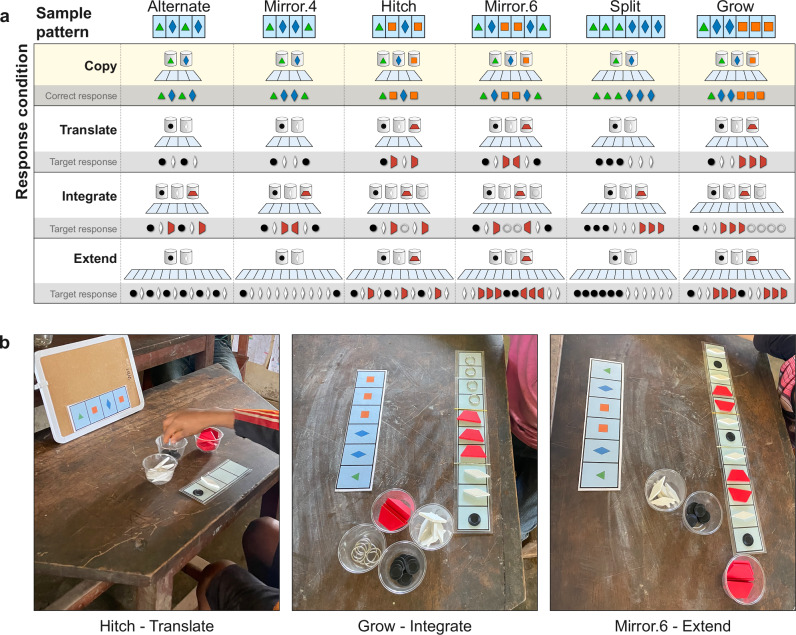
Pattern generalization task in Experiment 2. **a** We presented participants with each of six sample patterns composed of colored shapes (columns) and asked them to construct a response pattern that was as similar as possible, using a fixed set of response blocks and a response board of fixed length. After copying the sample pattern (top row), participants performed three generalization conditions (rows 2–4) in which copying was not possible: In making their response patterns, they used a set of response blocks that was different -- and sometimes larger -- than the set of blocks in the sample pattern. Example target responses are shown in the gray strip. **b** Tsimane' children performing generalization tasks.

Whereas children in Experiment 1 reproduced simple repeating patterns, children in Experiment 2 were asked to generalize complex patterns to new lengths and new stimuli, a better test of their ability to infer abstract logical structure. A new sample of 43 Tsimane’ children (ages 7–12 years) viewed each of six novel patterns, presented as static arrays of colored shapes, as shown in the columns of Fig. 4a. Whereas the patterns in Experiment 1 were all repeating (e.g., AABAAB), the patterns in Experiment 2 included some repeating patterns, some growing patterns (e.g., ABBCCC), and some mirror patterns (e.g., ABCCBA), as in prior work^71,80^. These stimuli provided a broader test of pattern understanding and, importantly, cannot all be composed by simply repeating a series of actions, learning the transition probabilities among items, or identifying repeating chunks (e.g., AAB). Rather, growing and mirror patterns follow abstract rules but do not repeat.

For each sample pattern, participants produced four response patterns, one for each of four experimental conditions. They started with the COPY condition, which required them to exactly reproduce the sample pattern. Whereas such reproduction was central in Experiment 1, here this condition functioned as a comprehension check on the task; In the few cases in which a participant failed to correctly make a copy of the sample pattern, subsequent responses to that sample pattern were excluded from analysis.

They then performed three generalization conditions (see the rows of Fig. 4a), which we constructed by varying the length of the response board (i.e., 4–12 spaces) and the composition of the response blocks — the ALPHABET (e.g., green triangles, blue diamonds, and orange squares). Starting from scratch each time, participants were asked to look closely at the sample pattern and make its “sibling,” a new pattern that was as similar as possible to the sample, given the items available and length required (for example target responses, see gray strips in Fig. 4). First, in the TRANSLATE condition, response length was the same as the sample, but the set of response blocks to choose from was different. For example, given an alternating sample pattern , participants were asked to make a response (also of length four) using only black and white response blocks (with different shapes as well; e.g., ). Participants thus had to generalize the abstract structure of the sample pattern to these novel stimuli^71,75^.

Second, in the INTEGRATE condition, a new block type was added to the response options, and the response board was lengthened (by 2–4 spaces). For example, given the same pattern , participants were asked to make a response pattern of length six using black, white, *and* red blocks (each with a distinct shape). Importantly, in this task, the sample pattern provided no information about how to use a third block type, which children could do systematically or randomly, many times or just once (but no less than once: Children were required to use at least one of each available block type).

Finally, in the EXTEND condition, we asked participants to make a response pattern of length twelve, using the same set of response blocks used in the Translate condition. By extending the response length in this condition, we dramatically increased the number of possible response patterns (i.e., 4096 or 531,441 possible patterns depending on alphabet size), allowing us to better observe any underlying regularity. We note that the response blocks differed from the sample blocks in all three generalization conditions (not just in the Translate condition), requiring that participants translate *and* integrate in the Integrate condition and that they translate *and* extend in the Extend condition. As in Experiment 1, responses in all three generalization conditions were under-determined by the data we provided (i.e., 4-6 items), as each sample pattern (e.g., ABBA) could reflect a wide variety of possible algorithms.

Participants’ response patterns were varied but highly structured. Collectively, participants made 242 unique response patterns, with the modal response accounting for as much as 98% and as little at 12% of responses to a given pattern (see Supplementary Fig. S4 in *Supplementary Information* for the full set of responses). Responses also varied within participants, whose response patterns had a unique structure in 92% of trials.

To analyze the accuracy of participants’ response patterns, we first compared them to a predetermined set of canonical target responses which share algorithmic structure with the sample patterns, examples of which are shown in Fig. 4a (see Supplementary Table S1 in *Supplementary Information* for full list). In most cases, these target responses were superficial variations on a single abstract pattern. For example, for the Mirror.4 pattern , we defined two target responses in the Translate condition —  and  — but both of these responses correspond to a single abstract pattern with structure ABBA. Only in the Extend condition, with its length-12 responses, did the number of these unique target response patterns exceed two. These patterns served as a conservative baseline for estimating how well participants’ responses reflected the structure of the sample patterns. We quantified accuracy as the edit distance (i.e., Damerau-Levenshtein distance) from each response pattern to the nearest target response; smaller distance reflects greater similarity.

**Fig. 5:**
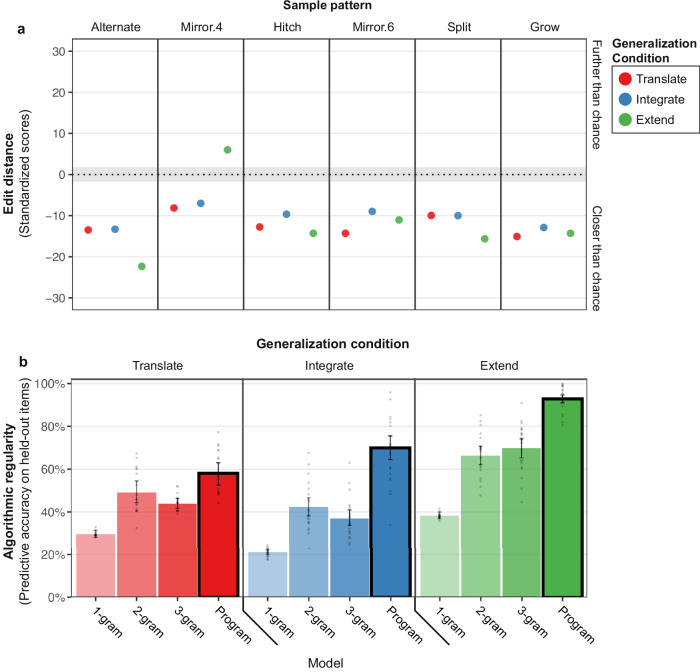
Results of Experiment 2, in subset-knowers only (*N *= 20). **a** Edit distance from nearest target answer for every sample pattern (columns) and generalization condition (colors): Higher points show larger mean edit distance. Specifically, Y-axis shows Z-scores derived from chance distribution, such that a point at Y = − 10 is 10 standard deviations closer than expected by chance (dotted line). The gray band shows 95% confidence interval around chance. **b** Results of generative Bayesian modeling, averaged by generalization condition. Bolded bars show how often the most likely program correctly predicted the (masked) elements of each response, points show individual participant values, and error bars show bootstrapped 95% confidence intervals. Lighter bars show the same measure for n-gram models, whose predictions depend only on associations among neighboring items.

Figure 5 a shows standardized mean edit distances for each generalization condition and sample pattern (as a proportion of pattern length). Edit distance was equal to zero in 45% of critical responses, meaning that participants produced one of the target patterns exactly. Other responses differed from the nearest target pattern by 1–6 insertions, deletions, and/or swaps (mean = 1.73 edits). To compare these observed edit distances to chance, we shuffled the positions of items within each response pattern and recomputed the shortest edit distance ten thousand times, yielding a chance distribution that defines the Y-axis of Fig. 5a. As that figure shows, for 17 of 18 sample patterns and conditions, participants’ responses were reliably closer to the target patterns than would be expected by chance, in both full-counters and subset-knowers (*ps* < . 001; see Supplementary Table S2 in *Supplementary Information* for detailed results), often by more than 10 standard deviations: Response patterns shared algorithmic structure with the sample patterns. The exception in both subgroups was the Mirror.4 sample pattern in the Translate condition, in which participants often made alternating response patterns rather than mirror-symmetric patterns, which suggests either a bias toward alternation or an order effect (as this pattern immediately followed the alternating pattern). Mixed-effects regression models showed that edit distances were not significantly different across ages (*t*(39.08) = 0.99, *p* = . 331, *β* = . 13, 95%*CI*[ − . 12, . 39], *N *= 44) or between numerical knower levels (*t*(35.19) = 1.72, *p* = .094, *β* = .28, 95%*CI*[ − . 03, . 60], *N *= 44), but were significantly smaller for participants with greater schooling, even controlling for these other variables (*t*(37.75) = − 2.72, *p* = .010, *β* = − . 23, 95%*CI*[ − .39, − . 07], *N *= 44; see Supplementary Fig. S2 in *Supplemental Information*).

To test the specificity of participants’ responses to each sample pattern, we also computed the edit distance of each response pattern to the nearest target response for each of the *other* comparable sample patterns (i.e., the ones participants were not looking at). Overall, responses were significantly more similar to the given pattern than to the other patterns (*p*<.001; see *Supplementary Information*: Response specificity).

As in Experiment 1, we also used a program-learning model to analyze individual responses. Performance was high even in the youngest participants (i.e., 7 years: 75%) and those with the least formal schooling (i.e., 1 year: 80%) and neither variable had a statistically significant effect on algorithmic regularity, even when each was the sole predictor (*ps* > .150; see Supplementary Fig. S2 in *Supplemental Information*). As shown in Fig. 5b, this program learning model predicted responses better than unigram, bigram, or trigram models, yielding 60–80% accuracy on masked tokens even among subset-knowers. Note that this program learning model did not see the sample pattern, but only predicted a masked token from the other tokens in each response pattern. This finding shows that participants’ responses cannot be explained by random responding or simple associations among neighboring items. Notably, the advantage of the program model was most clear for the INTEGRATE and EXTEND conditions, which required children to not only translate from one set of stimuli to another, but also to generalize the sample patterns to larger alphabets and greater lengths. Therefore, the regularities in participants’ response patterns cannot be attributed to simple heuristics (like imitation or substitution), nor can they be attributed to explicit instruction, as participants received no verbal description of the sample patterns or feedback on their responses. Rather, participants’ responses are best characterized as the products of program induction.

## Discussion

Humans are unique in the animal world for inhabiting a wide variety of environments and developing a rich array of cultural tools and practices^25^. Despite enormous cross-cultural diversity, children reliably learn the languages, skills, and conventions of their environment, starting at birth^27,66^. Some of these abilities benefit from instruction, but many do not, leaving children to infer implicit rules from observation alone. To clarify the cognitive mechanisms that underlie these extraordinary learning abilities, here we tested children in a domain unlikely to be the target of either cultural transmission or innate, domain-specific learning mechanisms. Across two experiments, children from two distinct cultures viewed novel sequences of actions and arrays of objects - short patterns that could reflect a broad array of possible algorithms. Despite the unfamiliarity of the task and the small amount of data they received, participants successfully generalized the observed patterns to new stimuli, new alphabets, and new lengths. Our measures of accuracy show that their response patterns reflected the abstract structure of the sample patterns they observed. Our computational modeling results show that this ability is likely driven by program induction: Even when they deviated from target responses, participants’ response patterns were highly algorithmic—better explained by compositional rule-learning than simple heuristics or statistics. These findings suggest that human skill learning can be understood as a form of algorithm discovery: Presented with small amounts of data, children across cultures induce the abstract logical structure therein, constructing ad-hoc explanatory theories without instruction, feedback, or knowledge of other common algorithms. This flexible, domain-general learning mechanism can help to explain how children can learn any number of cultural practices, social norms, and physical skills across a wide variety of natural and cultural environments.

These findings clarify the mechanisms by which naive learners infer the structure of novel patterns, as found in previous studies^66,71–77^. Many of the patterning tasks that have been used can be solved in principle using strategies that fall far short of program induction, leaving its role in question. When using a consistent set of stimuli, children could, in principle, recognize, copy, and even extend patterns to new lengths using associative learning alone, reproducing the transition probabilities between types (e.g., B always follows A; C always follows two Ds;^117^). To generalize a pattern to new stimuli, as in our TRANSLATE condition and some studies of infant rule-learning^66,67^, learners need only convert one alphabet into another (e.g., A → X, B → Y), without any need to infer the patterns’ compositional structure. By contrast, participants could not rely on simple translation in our INTEGRATE condition, in which the response alphabet was *larger* than the sample alphabet by design. Rather, to succeed in this condition, participants needed to generalize the compositional structure of the sample pattern to the larger set of response options (e.g., from 2 to 3 block types). And they did: Response patterns in this condition were far closer to the sample patterns than would be expected by chance and were better described by a program-induction model than by simple n-gram models. These results provide evidence that children’s understanding of novel patterns was not driven by statistical learning or simple heuristics, but by a form of program induction, which may be active very early in life in children across cultures^9,11^. On this account, patterning ability may covary with other cognitive abilities, like mathematical reasoning and problem-solving, in part because these abilities are all driven by a program-induction engine that operates across domains and whose performance varies over development and across individual learners.

To be clear, we do not wish to claim that children perform program induction in the specific way we have implemented here; Alternative implementations relying on different primitives and different compositional constraints may account for behavior equally well^60^. Nor do we wish to claim that simple association learning (like transition probabilities) is irrelevant to this kind of learning - it is not. Rather, the central claim is that when children encounter new data, they appear to rapidly induce not just the regularities therein, but also the rules, performing an inferential process that is analogous to inducing programs in a language of thought. However it is implemented, this process of program induction can in principle provide a powerful and highly general learning mechanism that extends well beyond imitation and association. An important question for future work is how people search large (or infinite) hypothesis spaces like those presented by our patterns and how the set of conceptual primitives in the language of thought shapes what people infer in a particular domain^13,126–130^.

Many of our participants were young, lacked extensive schooling, and understood little about common algorithms like counting, but they all were practiced in pattern recognition, including in language, physical reasoning, social inference, and a variety of cultural practices involving patterns. For example, Tsimane’ children often participate in fishing, harvesting, and preparing food^27^. Such activities provide them with ample opportunity to infer the implicit rules that govern their environment, starting from birth, and may therefore improve any extant program-induction abilities, even if they are innately available in some form. In this way, even passive participation in cultural groups may hone children’s ability to induce implicit structure from data. These experiences, along with changes in general cognitive capacities like working memory and executive control, may explain why older children were generally more accurate and algorithmic in our tasks. Algorithmic abilities may also be influenced by formal and informal instruction^91,131,132^, in which teachers and caregivers make explicit the algorithms underlying cultural skills like adding fractions, tying shoelaces, or building canoes. Although our analyses showed limited effects of formal schooling, even small amounts of schooling can have large effects on children’s performance in some tasks, including generalization tasks^131,133,134^. Future work should more closely examine the effects of education on the type and extent of generalization in pattern-induction tasks.

In principle, cultural experience could allow children to reproduce familiar patterns without inferring their abstract structure, but this possibility cannot explain participants’ performance in our experiments, for three reasons. First, if the colors and shapes of our stimuli (e.g., red circles) were familiar to participants from other activities - and they likely were - participants might try to generalize from those activities to our tasks. However, assuming that these shapes/colors played different roles in those activities, this prior experience would be a source of noise in our experiment, potentially interfering with participants’ ability to correctly reproduce or generalize the given pattern, rather than facilitating it^135^. Second, some cultural activities could share an abstract algorithmic structure with our sample patterns, despite superficial differences in appearance. For example, patterns like Sort and Split may be familiar to children who have distributed objects among two parties and patterns like Mirror.4 may be abstractly analogous to balancing objects on a scale. Yet, such similarities in abstract structure cannot explain success in our task because participants observed the form only - the abstract structure must be inferred in order for any structural analogies to be made. Finally, whereas it is unlikely that our participants had already encountered the specific sample patterns we used, it is even less likely that they had already generalized those patterns as they did in Experiment 2, translating them to new stimulus sets, larger alphabets, and greater lengths. In this way, children’s everyday cultural experience likely facilitated their performance in our tasks, but only insofar as it improved their pattern induction abilities in general.

Although program induction may support many human-unique cognitive abilities, it may not be unique to humans. Many non-human animal species show evidence of flexible learning, abstraction, and generalization^136–139^, even in domains unlikely to be the target of natural selection^140^, and at least some of this learning may reflect program induction. For example, although slower than humans, rhesus macaques learned the latent recursive structure in novel patterns and generalized it to new stimuli^141^. Whereas some accounts attribute humans’ extraordinary cognitive abilities to qualitative differences in learning mechanisms^14,53,62^, others attribute them to *quantitative* differences in information processing capacity^142^. In model systems, differences in memory alone can have profound impacts on what can be learned, holding constant the amount of data and the inferential mechanisms. In this way, program induction could be fundamental to learning in humans and non-human animals alike, despite dramatic differences in their cognitive abilities.

Children’s striking cognitive abilities in domains like natural language, causal inference, and numerical and physical reasoning have lead many theorists to posit a variety of learning mechanisms specific to those domains and to conduct many thousands of studies seeking to characterize them. Alternative accounts posit that learning across domains may largely be driven by a common set of inductive processes in which children construct and compare competing theories on the basis of the data they observe^11,130,143^. Findings from formal learning models show that such learning is in principle possible: Given only a small set of domain-general logical primitives, an idealized learner can induce algorithmic structure in domains like counting^51^, family relations^144^, magnetism^130^, language^48^, and geometry^52^. The present results clarify how human learners across disparate cultures can infer even arbitrary patterns in a novel domain without instruction, knowledge of common algorithms, or large amounts of data. To be clear, these findings do not rule out domain-specific learning mechanisms, which may guide learning in domains like spatial reasoning, social relations, and natural language^145^. However, they suggest that such mechanisms are either complemented by more domain-general mechanisms or are simply not very domain-specific. For example, in principle, participants could have performed our tasks using the same learning mechanisms they used to learn language, inferring the implicit rules structuring each visual pattern just as they inferred the implicit syntactic rules structuring Tsimane’, Spanish, or English. However, if these learning mechanisms apply not just to natural language but also to any meaningless pattern of shapes or sequence of actions, then they may be better characterized as mechanisms for pattern learning in general, rather than for language learning per se^146,147^. Likewise, in the domain of number, scholars have long attributed children’s counting abilities to innate neural mechanisms, in part because of the difficulty of the computational challenge such algorithms pose to the learner^101^. This argument is undermined by our results, which show that children can learn a variety of such algorithms from a single observation, even before they have learned to count. We suggest that many of the specialized skills people acquire in a given setting — from counting to cultivating crops to parallel parking — can be understood as algorithmic abilities learned via learning mechanisms that are broadly shared across domains. In this way, program induction may be central to both our cognitive flexibility and cultural diversity, allowing people to discover and generalize the latent algorithmic structure of their natural and cultural environment, whatever it may be.

## Methods

### Ethics and inclusion

This research with indigenous Tsimane’ communities included local researchers in the design and implementation of the study. They included two local Bolivian researchers with extensive knowledge of Tsimane’ culture and language, who were consulted on task design, community selection, and best research practices for Tsimane’ communities. We also worked with a small team of native Tsimane’ research assistants, who contributed to study recruitment, implementation, translation, and compensation. These contributors are included in the acknowledgments and have full access to the data and analysis scripts, which are freely available. The research concerns fundamental cognitive processes across human groups and so is relevant to people broadly, rather than to any specific community, including Tsimane’ communities. Roles and responsibilities were agreed among collaborators in advance. We did not discuss capacity building with local researchers. The experiments were not randomized. We conducted the study both in Tsimane’ communities in Bolivia and in US American communities in California. Limiting the study to the setting of the primary researchers would have limited the generalizability of the findings and the ability to characterize the relation between numerical knowledge in algorithm induction.

The study was approved by the UC Berkeley ethics committee (Protocol #2018-06-11209) and by the appropriate local body, *El Gran Consejo Tsimane’* (The Tsimane’ Grand Council), which oversees research in Tsimane’ communities. The research does not present any known risk of stigmatization, incrimination, discrimination or other personal risk to participants. Nevertheless, to mitigate risks to the health and safety of researchers and participants alike, fieldwork was planned, coordinated, and overseen by experienced field researchers and local experts with intimate knowledge of the local language, culture, and landscape (see below). We also wrote and implemented a COVID-safety protocol. In our citations, we have taken into account local and regional research relevant to our study.

### Mitigating cultural bias

We took several measures to limit any effects of researcher bias on our cross-cultural findings in both experiments. First, our Tsimane’ research was conducted in partnership with el Centro Boliviano de Investigación y Desarrollo Socio Integral (CBIDSI), a Bolivia-based NGO specializing in the study of Tsimane’ health and culture, whose expertise informed the selection of villages, participant recruitment, informed consent, compensation, and task design. During Tsimane’ data collection, experimenters from US universities were accompanied by native Tsimane’ research assistants who acted as the primary touch points with participants and provided them with instruction in their native language. Second, to mitigate misunderstanding of the tasks, we provided demonstrations, practice trials, and comprehension checks to evaluate participants’ understanding behaviorally before performing critical trials. Third, our outcome measures were standardized across individuals and groups, did not include informative feedback, and focused on observable features of participants’ behavioral responses, leaving little room for interpretation. The study was not preregistered because of the nature of this type of fieldwork, in which neither the sample composition nor the final task design can be decided in advance.

### Experiment 1

#### Participants

Tsimane’ children (*N *= 72; 30 female; ages 3–13 years; mean age = 7.5 years, mean schooling = 1.4 years) were tested in their villages along the Maniqui river of lowland Bolivia in Summer 2019. The research team included US Americans, Bolivian academics, and native Tsimane’ experimenters. Villages were selected according to their accessibility from San Borja, in consultation with local experts. Tsimane’ participants consisted of volunteers presenting themselves at the village schoolhouse. No statistical method was used to predetermine sample size: The number of participants was determined by the duration of the field trip and testing conditions (e.g., weather, travel time to villages, etc.) As many Tsimane’ adults do not read or write, all consent procedures and instructions were conducted orally by a translator who grew up in a local community, as approved by the UC Berkeley IRB. The Tsimane’ translator explained aloud in Tsimane’ the purpose, risks, benefits, duration, and voluntary nature of our study, as well as the age range of interest. Potential participants were encouraged to ask questions. Parental consent to child participation and data usage was documented by the research team. Given parental consent, we showed children the experimental stimuli and asked them if they would like to play a game with us using those stimuli. A positive response constituted assent, and researchers intermittently reassessed assent during testing. Parents were invited to sit nearby participants for their comfort, but to abstain from assisting in any way with the tasks. Parents and children were compensated with small gifts, regardless of their performance or completion.

US children (*N *= 24; 14 female; ages 3:1–5:5; mean age = 4.4 years, mean schooling = 1.4 years) were tested in preschools affiliated with UC Berkeley in Berkeley, California. The sample size was determined by the availability of these preschools prior to fieldwork. Parents were provided with a written description of the study’s purpose, risks, benefits, duration, and voluntary nature. A subset of these parents provided advance written consent for their child’s participation and data use. Of these children, participants were chosen in consultation with the preschool teachers on the basis of age and history of research participation (to limit their frequency of participation). US child assent was obtained as among Tsimane’ children. Participants were gifted a sticker regardless of performance or completion. Gender was assessed by the experimenter, but neither sex nor gender was analyzed, as they were not of theoretical interest.

#### Schooling

We assessed participants formal schooling by asking parents to report the number of years the child had attended school. We note that this provides an imperfect measure of education among the Tsimane’, in part because Tsimane’ schooling is generally less standardized than in industrialized societies. For example, many Tsimane’ school teachers lack pedagogical training, and many children attend school only irregularly. Children’s basic counting abilities, described below, provide a behavioral proxy of schooling.

#### Numerical tasks

Participants’ performed two tests of their basic numerical abilities. In the verbal counting task, we asked children to count up from one (in whatever language they preferred). Because children were sometimes shy with the experimenters, we conducted a non-numerical warm-up exercise beforehand: An experimenter asked participants to name some of their friends and to list some of their favorite foods. If participants still struggled or appeared shy during counting, the experimenter recited the first two number words for them and asked them to continue (e.g., “uno, dos, ...”). The same procedure was used to test the highest count in both Spanish and Tsimane’ and the higher count was recorded as a participant’s highest verbal count.

We then used the Give-N task to test participants’ numerical knower-level^92,148^. In each trial, an experimenter placed 12 pebbles in a pile near the participant and asked them to move N to another location on the table, using the number word in Spanish and in Tsimane’ (e.g., “Dame tres, chibin”). Participants responded to each of the number words 1–8 in ascending order twice. To avoid giving informative feedback unintentionally, we asked participants to press a plastic button when satisfied with their response. The experimenter then recorded the cardinality of the response set and moved the response pebbles back into the set of 12 for the next trial. We classified Full-counters as those who made one or no errors for sets of 5-8 objects across both rounds; Others were classified as Subset-knowers (*N *= 57).

#### Algorithm tasks

Participants were seated at a test table across from the experimenter. Participants were told that the experimenter (Tsimane’ participants) or a puppet named Beary (US participants) had a job that needed to be done and would sometimes need help from the participant. For each of three algorithms - Sort, Double, and Hitch (in that order) - the experimenter presented four iterations of the algorithm while describing these actions aloud, specifying the number and color of each ball placed in each bin (without labeling the bins; see Fig. 2). After observing four repetitions of the given pattern, the participant was asked to continue the pattern: They drew colored balls from one or two transparent source bins, and freely placed them in the response bins 50 s while an experimenter recorded their responses without informative feedback.

In the **Sort** task, the experimenter drew red and blue balls from a source container and placed them in different bins, alternating bin and color at each step (e.g., red-left, blue-right, red-left, blue-right). The starting color and the specific assignment of color to the side varied across participants. The **Double** task differed from the Sort task in that two of one color were placed at each step (e.g., “One red, two blue, one red, two blue”). The **Hitch** task followed the same logic as Sort, except that a blue ball, chosen from a bin of only blue balls, was carried along with each response (as if “hitching” a ride; e.g., “One red and one black, one blue and one black...”). In all tasks, the starting color, the assignment of color to side, and the assignment of color to number was varied across participants.

#### Analysis of accuracy

We quantified how accurately participants reproduced various aspects of a given sample pattern, based on their use of color, number, and response bin, yielding five unique measures of performance, as shown in Fig. 3a. The **Color-Bin** measure represents how consistently participants placed balls of a given color in the same bin. **Number/Bin** is based on the number of balls (of any color) participants placed into a given bin before switching bins (e.g., 1 left, 2 right, 1 left...) and **Number/Color** is based on the number of balls of a given color participants used (in any bin) before switching colors (e.g., 1 red, 2 blue, 1 red); higher scores reflect higher accuracy (i.e., proportion correct number). The correct values for each of these measures differed across tasks, such that good performance required attending to the specifics of each sample pattern.

The two other measures were task-specific. Unlike other tasks, Double required alternating the number of balls per bin (e.g., 1 left, 2 right, 1 right...); **Number-Alt** measures the consistency of this numerical alternation. Hitch was unique in that it required participants to place two colors in each bin (i.e., red+black, blue+black, red+black...); the color** combo** measures how often participants in Hitch correctly placed two different colors in the same bin before switching bins.

To operationalize chance performance, we computed each of these measures on 10,000 versions of the data in which the correspondences between bin, color, and step were randomly permuted within each response pattern. We then z-scored this permuted data by participant and sample pattern and used this standardized scale to define the Y-axis in Fig. 3a, such that a score at *Y* = 15 is fifteen standard deviations above the mean score in the permuted data.

In principle, participants could perform above chance even while producing the same pattern in response to different sample patterns. We therefore tested the specificity of participants’ responses to each sample pattern, using edit distance (i.e., Damerau-Levenshtein distance) as a measure of pattern similarity. This technique computes the minimal number of deletions, additions, or substitutions required to transform a response pattern into the sample pattern. Results showed that in both groups and numerical-knower levels, participants’ response patterns were more similar by this metric to the given sample pattern than to the other sample patterns (*ps* < . 001; see *Supplementary Information*: Response Specificity for detailed results). In sum, responses were both accurate and specific to the given pattern.

#### Bayesian Inference Model

We used a Bayesian inference model based on the one described in ref. ^48^ to infer the program most likely to have produced each response pattern, regardless of its accuracy (full model available osf.io/eawkh). Like many others, this model characterizes learning as constructing and evaluating hypotheses: Mental programs in a language of thought^13,44,46,52,59,61,62,123,124,149^. Programs are constructed from a small set of conceptual primitives, including logical operators like *and*, *or*, and *if*, arithmetic operators like *plus*, *times*, and *count*, and list functions like *append* and *insert*. The set of primitives is shown in Table 1. Additional symbols were included for the terminals in the alphabet, empty string, and argument to the function being defined ("*x*”). These primitives are intentionally domain-general and are inspired by models of human behavior in a variety of domains, including syntax, counting, and kinship, among others (e.g., refs. ^48,51,144^).

**Table 1:** Primitive operations used in the Bayesian inference model

Primitive	Input → Output	Gloss
*length*	String → Integer	Return the number of items
*head*	String → String	Return everything but the last item
*tail*	String → String	Return everything but the first item
*emptystring*	${{\varnothing }}\to$ String	Return an empty string
*append*	String, String → String	Append two strings
*reverse*	String → String	Reverse the order of items
*repeat*	String → String	Return a string twice in a row
*count*	String, String → Integer	Number of times a substring appears
*recurse*	String → String	Evaluation of the current hypothesis
*plus*	Integer, Integer → Integer	Add two integers
*minus*	Integer, Integer → Integer	Subtract one integer from the other
*times*	Integer, Integer → Integer	Multiply two integers
*empty*	String → Boolean	Evaluate whether the string is empty
*equal*	String, String → Boolean	Evaluate whether two strings are equal
*and*	Boolean, Boolean → Boolean	Logical conjunction
*or*	Boolean, Boolean → Boolean	Logical disjunction
*not*	Boolean → Boolean	Logical negation
*if*	Boolean, Integer, Integer → Integer	Conditional for integers
*if*	Boolean, String, String → String	Conditional for strings

We then defined a probabilistic context-free grammar^150^ that assigns a prior probability to any composition of primitives (see Fleet github), with a preference to use a smaller number of these cognitive operations, following empirical results showing that this bias accurately models people’s rule-based concept learning^60,149,151^. In Bayesian inference, the belief (posterior probability) in any candidate hypothesis is proportional to the product of its prior probability, which favors simplicity, and its likelihood (how well it predicts the data). Here, the data are strings that encode the sequences of actions that participants made (i.e., the color and placement of each ball at each step). As described above, we modeled response sequences with one masked item for each token (position) in the sequence. For example, if participants produced the response sequence ABAB (where “A” and “B” correspond to putting particular objects in buckets (Experiment 1) or placing particular response tokens (Experiment B), then the model would, separately, be given sequences with each place masked (by “#”): ABA#, AB#A, A#AB, #BAB. For each, the likelihood was defined to sum over all possible values that the masked character could take:

1 $$P(ABA\#| H)=P(ABAA| H)+P(ABAB| H)+\ldots$$

where the sum runs over all allowed alphabet tokens, and each individual term used the insert-delete likelihood from ref. ^48^. This Bayesian inference model was used to predict the most likely value of *#* by Bayesian model averaging over hypotheses *H*. Thus for any token *x*,

2 $$P(ABAx| \,{{{\rm{Program}}}})={\sum }_{H}P(H| ABA\#)\cdot P(ABAx| H)$$

where *H* are program hypotheses, found using the Markov-Chain Monte-Carlo techniques in ref. ^48^. For each masked position in each data string, we ran a search for 30 min and stored the top 100 programs found for each masked position. To efficiently explore the hypothesis space while avoiding local maxima, we used Markov-Chain Monte-Carlo with parallel tempering, with 6 chains running at a ladder of different temperatures^152^. A chain was restarted from the prior if it failed to reach a new maxima after 1 million steps. The Bayesian model average accuracy, *P*(*ABAx*∣Program Model) for the subject’s observed answer *x*, provides the Y-axis in Figs. 3c and 5b.

For comparison, we also modeled these masked responses using simple *n*-gram models, which attempted to predict the masked element given only the previous two elements of the response (trigram), given only the preceding element (bigram), or simply from the base rate of elements (unigram), a conservative measure of random responding. We used lambda smoothing to avoid assigning zero probability to unseen n-grams, although we note that these models generally do not perform well because a short segment of a subject’s response, like that in ABA#, does not provide much information about the sequential probabilities.

### Experiment 2

#### Participants

We tested another sample of Tsimane’ children (*N *= 43; 21 female; age range: 7–12 years; mean age = 9.3 years, mean schooling = 3.6 years) during a second trip to Bolivia in the Summer of 2022. As in Experiment 1, we followed experimental protocols approved by the Institutional Review Board at the University of California, Berkeley and by El Gran Consejo Tsimane’ (The Tsimane’ Grand Council) and implemented measures to limit experimenter bias. We received assent from all participants and consent from their legal guardians on participation and publication of video, photo, and audio recordings, as in Experiment 1. Gender was determined by the experimenter, but neither sex nor gender was analyzed, as they were not of theoretical interest.

#### Schooling

The amount of formal schooling was assessed as in Experiment 1.

#### Numerical tasks

Procedures were the same as in Experiment 1, except in the Give-N task, in which we tested each of the number words (1–8) once increasing order and then again in a random order chosen by the experimenter. Again, we classified Full-counters as those who made one or no errors for sets of 5–8 objects across both rounds; Others were classified as Subset-knowers (*N *= 20).

#### Algorithm tasks

After completing the numerical tasks, participants performed a series of algorithm tasks composed of six patterns of colored shapes, as shown in Fig. 4: Alternate (ABAB), Mirror.4 (ABBA), Hitch (ABCB), Mirror.6 (ABCCBA), Split (AAABBB), and Grow (ABBCCC). The sample patterns consisted of 4 or 6 colored shapes (e.g., green triangles, blue diamonds, orange squares, etc.), which appeared on a printed card. These cards were oriented horizontally (i.e., as a single row of colored shapes) and positioned in the center of the testing table, where they remained visible to participants throughout the relevant trials. In a brief warm-up task, we presented participants with the Alternate (ABAB) and Mirror.4 (ABBA) cards simultaneously and asked them if the two patterns were the same or different. If they were unable to identify differences between the patterns, the experimenter pointed to individual items of the cards, showing the participant where they were the same and where they were different. A small number of participants continued to characterize the cards as the same even after repeated explanations from the experimenter, and so testing was discontinued. All others performed four trials for each pattern, one for each generalization condition, in which they selected colored blocks from a given response set and placed them onto a printed response board with a fixed number of empty spaces.

To begin, in the Copy condition, participants were given a response set and response board like those pictured in the printed sample pattern and were asked to make their response board the same as the sample. For example, if the sample consisted of two green triangles and two blue diamonds, participants received a response board of length four, a cup of green triangle blocks, and a cup of blue diamond blocks. Each cup contained 10 colored blocks, far more than were needed. These copy trials served as a comprehension check, testing whether participants understood the intended relationship between the printed sample pattern, the colored blocks, and the response board. Participants who did not make a perfect match on their first attempt were asked to repeat this condition.

Having successfully copied, participants advanced to the three generalization conditions. Since copying was not possible in these trials, participants were asked to make a “sibling” of the sample pattern, using the set of response blocks provided. This wording avoided the use of words like “same,” whose meaning varies across cultures and contexts. Responses that did not include all available block types (3%) were excluded from analysis. Specifically, in Translate trials, participants made a response pattern of the same length as the sample, but using a new set of response blocks with different colors and shapes (e.g., black circles and red trapezoids). Participants who did not translate correctly on their first attempt were asked to repeat this condition, but only initial responses were analyzed. Then, in Integrate trials, another unfamiliar block type was added to the response set and the length of the response board was increased accordingly (from 4 to 6 spaces or from 6 to 8, 9, or 10 spaces). Finally, in the Extend trials, participants used the same set of blocks as in Translate to construct responses of length twelve. Note that in all three of these critical conditions, all response objects were different from those depicted in the sample pattern. Participants received no feedback on their responses.

#### Analysis of accuracy

In Experiment 2, we measured accuracy by computing the edit distance (i.e., minimum number of swaps, deletions, or insertions) between each response pattern and the nearest target response for each task (see Supplementary Table S1 in *Supplemental Information* for a complete list). To compare these observed edit distances to chance, we recomputed the group mean after each of 10,000 permutations, in which we randomly scrambled the positions of blocks in participants’ responses. This chance distribution defines the Y-axis in Fig. 5a. Our measure of edit distance is therefore conservative both because our set of target responses is not exhaustive, and because our permutations respect the marginal distributions of response blocks, incorporating any idiosyncratic preferences for specific colors or shapes that informed participants’ responses (e.g., a preference for orange squares). To perform well on this measure, participants had to do more than produce responses with algorithmic structure; Their responses had to reflect the specific algorithmic structure of the sample pattern they were given.

#### Bayesian Inference Model

In Experiment 2, we used the same model as in Experiment 1. Raw responses were recoded according to their ordinal position to allow participants to use any response block in any position. With this technique, inverted patterns like  and  both corresponded to the same underlying code, in this case: 1221.

### Reporting summary

Further information on research design is available in the Nature Portfolio Reporting Summary linked to this article.

## Supplementary information

Supplementary Information

Reporting Summary

Transparent Peer Review file

## Data Availability

All raw data are publicly available online at 10.17605/OSF.IO/EAWKH^153^.

## Code availability

All analysis scripts are publicly available online at 10.17605/OSF.IO/EAWKH^153^.
