# Supplementary material for: Children use algorithm induction to discover patterns in data
Source: Nat Commun. 2026 May 30;17:7017. doi: 10.1038/s41467-026-73029-9 (PMC13392036; doi:10.1038/s41467-026-73029-9)
Supplement: Supplementary file 1 — Supplementary Information [file 41467_2026_73029_MOESM1_ESM.pdf]

Algorithm induction in indigenous Amazonian children

Supplemental information

Benjamin Pitt (pitt@uchicago.edu), Elena Leib, David O'Shaughnessy,
Charlene Gallardo, Stephen Ferrigno, & Steven T. Piantadosi

March 19, 2026

Supplementary Methods

Table S1: **Target responses in Experiment 2.** For each sample pattern and generalization condition, edit distance was computed between participants' response pattern and these target patterns.

**Sample**	**Coded**	Copy	Translate	Integrate	Extend
**Alternate**	**1212**	1212	1212	123123	121212121212
**Mirror.4**	**1221**	1221	1221	123321	122112211221
**Mirror.4**	**1221**				122222222221
**Mirror.4**	**1221**				112222222211
**Mirror.4**	**1221**				111222222111
**Mirror.4**	**1221**				111122221111
**Mirror.4**	**1221**				111112211111
**Hitch**	**1232**	1232	1232	123242	123212321232
**Hitch**	**1232**				.2.2.2.2.2.2
**Mirror.6**	**123321**	123321	123321	12344321	123321123321
**Mirror.6**	**123321**				112233332211
**Mirror.6**	**123321**				123321321123
**Mirror.6**	**123321**				123321231132
**Mirror.6**	**123321**				123321132231
**Split**	**111222**	111222	111222	111222333	111222111222
**Split**	**111222**				111111222222
**Split**	**111222**				122222222222
**Split**	**111222**				112222222222
**Split**	**111222**				111222222222
**Split**	**111222**				111122222222
**Split**	**111222**				111112222222
**Split**	**111222**				111111222222
**Split**	**111222**				111111122222
**Split**	**111222**				111111112222
**Split**	**111222**				111111111222
**Split**	**111222**				111111111122
**Split**	**111222**				111111111112
**Grow**	**122333**	122333	122333	1223334444	122333122333
**Grow**	**122333**			1223331234	122333133222
**Grow**	**122333**				122333211333
**Grow**	**122333**				122333233111
**Grow**	**122333**				122333311222
**Grow**	**122333**				122333322111
**Grow**	**122333**				122333333333
**Grow**	**122333**				122233333333
**Grow**	**122333**				122223333333
**Grow**	**122333**				122222333333
**Grow**	**122333**				112223333333
**Grow**	**122333**				112222333333
**Grow**	**122333**				111222233333
**Grow**	**122333**				111222233333
**Grow**	**122333**				111222233333

Supplementary results

Response specificity

In addition to tests of accuracy, we also tested whether participants gave different responses to different sample patterns, using edit distance (i.e. Damerau–Levenshtein distance) as a measure of pattern similarity. All patterns were recoded into a common alphanumeric code that allowed easy comparison of abstract structure, independent of superficial differences (e.g. ABBA vs. BAAB).

In Experiment 1, we computed the edit distance between each response and each of the three sample patterns (up to the length of the response pattern). On average, distances were smaller to the given pattern than to the other patterns ($\beta = -.87, SEM = .04, p < .0001$, N = 97) and this overall effect obtained for each of the three sample patterns, both groups, and both numerical knower-levels ($ps < .0001$). As shown in Figure S1 (left), the exception among the pairwise comparisons was in the Sort task, in which participants' responses were no closer to the Sort sample pattern than to the Double sample pattern. As responding in this experiment was largely unconstrained, these differences in response patterns reflect different choices by the participants, not differences in task constraints.

By contrast, in Experiment 2, participants' responses were in part constrained by the design of the tasks, which differed in the size of the alphabet (i.e. 2 vs. 3 block types) and the required length of the response (i.e. 4 - 12 items). We therefore compared only those responses in the same *family* - that is - those that shared the same size alphabet and response length, in the same condition, which permitted comparison of each sample pattern to one or two others (see Figure S1, right). This approach avoids comparing patterns of different lengths and different alphabet sizes, which would artificially inflate the measure of specificity. As in Experiment 1, participants' responses in Experiment 2 were more similar on average to the given pattern than to the other comparable patterns ($\beta = -2.11, SEM = .07, p < .0001$, N = 44), and this effect obtained for four of the five sample patterns ($ps < .0001$). The exception was the sample pattern GBBG, to which participants gave responses that were significantly *farther* from the given pattern than to other patterns (e.g. GBGB; $p = .0002$).

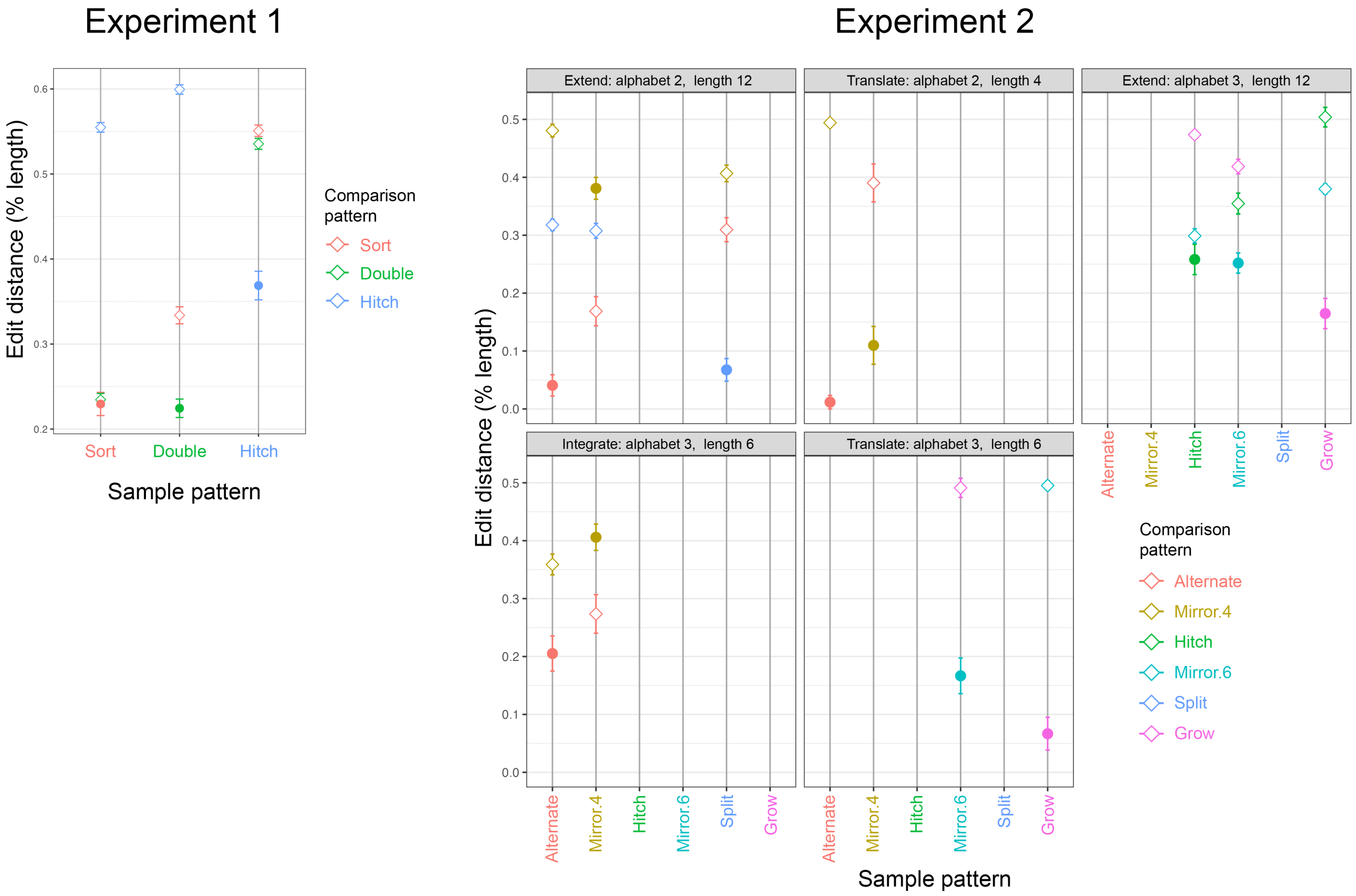

Figure S1: **Specificity of responses in both experiments.** In each vertical line, the solid point shows the mean edit distance of response patterns (as a proportion of their length) to the given sample pattern; Diamonds show the same measure to other sample patterns; Error bars show bootstrapped 95% confidence intervals Smaller edit distances indicate greater similarity. Plots for Experiment 2 (right) are faceted by family, where each family includes comparable tasks (i.e. tasks in the same generalization condition with the same size alphabet and response length).

Effects of age and schooling

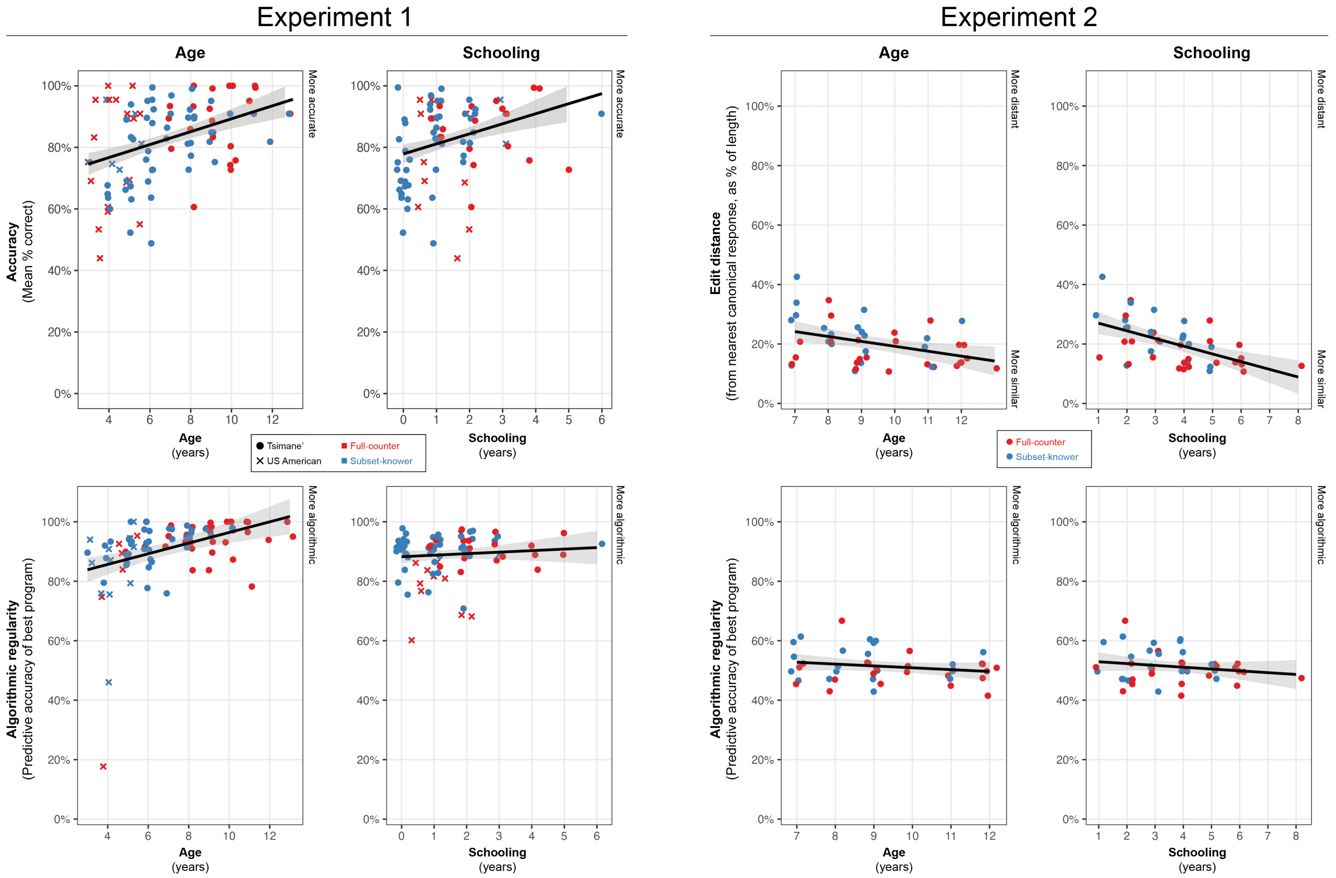

Figure S2: Participant mean accuracy and algorithmic regularity by reported years of schooling and by age in each experiment. Grey error bands show 95% confidence intervals around the regression line.

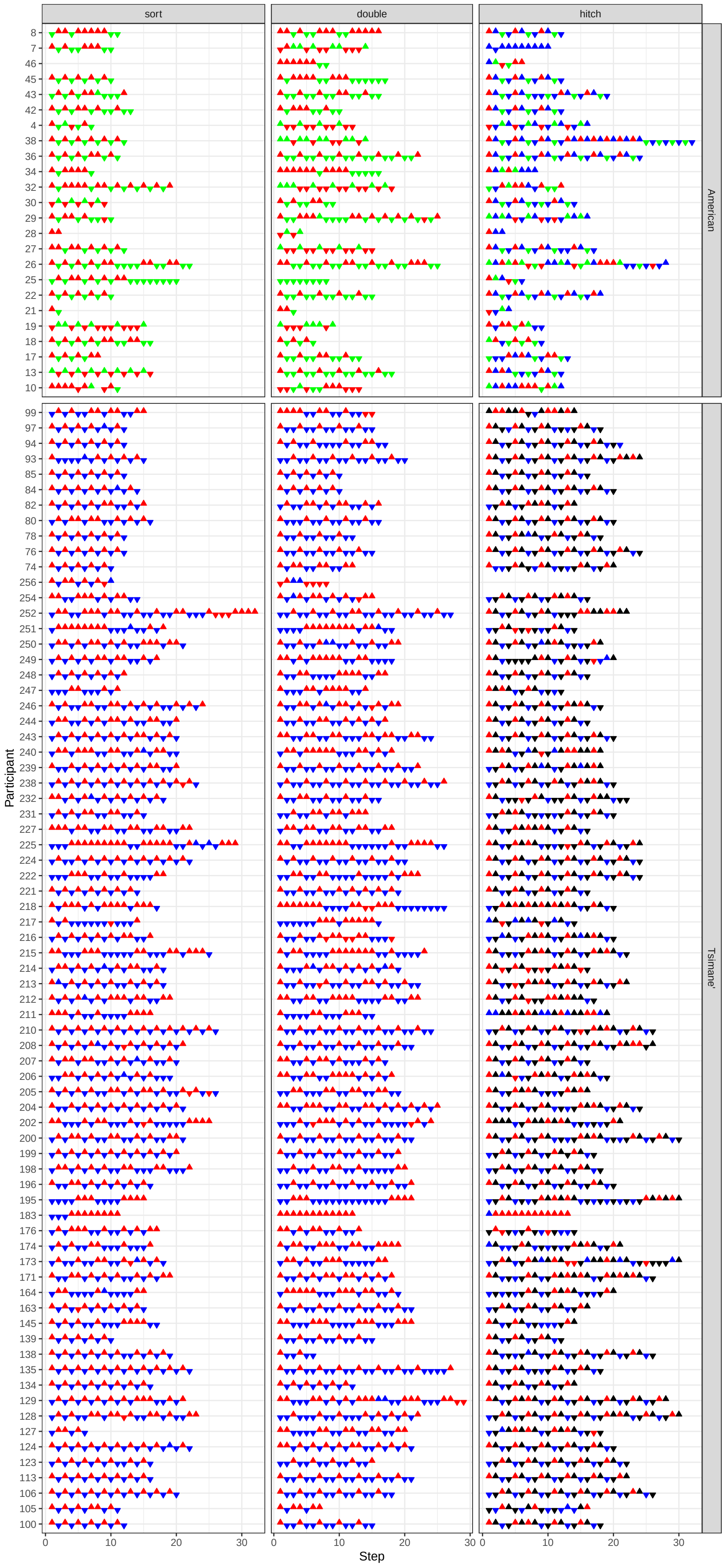

Figure S3: **All response patterns in Experiment 1.** Participants (rows) were asked to extend each of three sample patterns (columns). Upward triangles show balls placed in left bin; Downward triangles show balls placed in right bin.

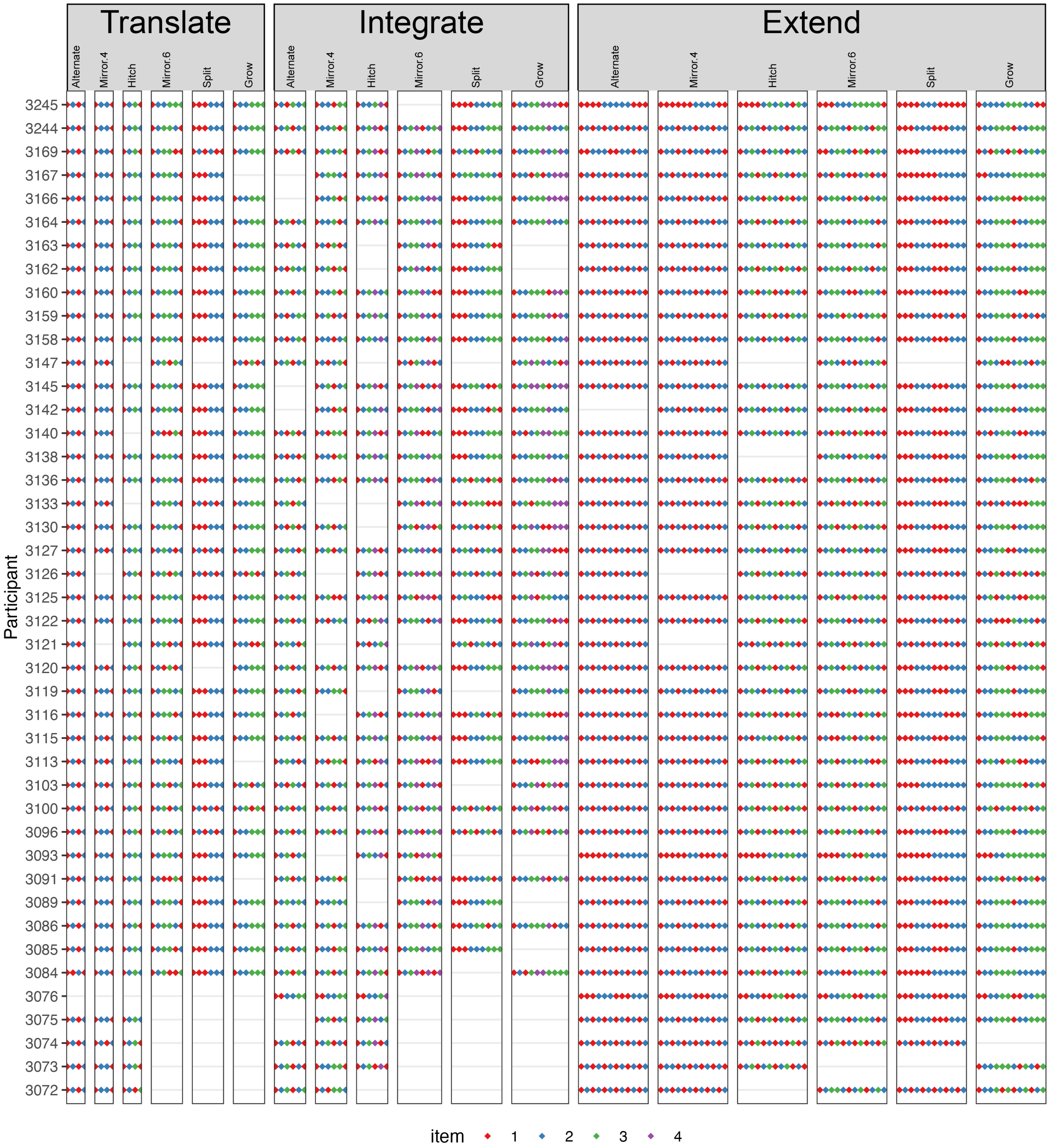

Figure S4: **All response patterns in Experiment 2.** Participants (rows) were asked to generalize each of six sample patterns (columns) in each of three generalization conditions (groups of columns). Colors represent ordinal position (not the literal color) of the response block.

Table S2: **Full accuracy results from Experiment 1.** Observed means were compared to means computed over 10,000 permutations.

Condition	Group	Algorithm	Measure	Mean	95\% CI	N	P
Subset	Tsimane’	sort	color_bin	0.98	[0.6, 0.64]	52	≤ 0.001
Subset	Tsimane’	sort	num_bin	0.65	[0.4, 0.49]	52	≤0.001
Subset	Tsimane’	sort	col_num	0.75	[0.44, 0.53]	52	≤0.001
Subset	Tsimane’	double	color_bin	0.98	[0.62, 0.65]	52	≤0.001
Subset	Tsimane’	double	num_bin	0.82	[0.67, 0.75]	52	≤0.001
Subset	Tsimane’	double	col_num	0.85	[0.72, 0.79]	52	≤0.001
Subset	Tsimane’	double	num_alt	0.41	[0.18, 0.28]	52	≤0.001
Subset	Tsimane’	hitch	color_bin	0.94	[0.69, 0.73]	51	≤0.001
Subset	Tsimane’	hitch	num_bin	0.73	[0.25, 0.33]	51	≤0.001
Subset	Tsimane’	hitch	col_num	0.99	[0.81, 0.87]	51	≤0.001
Subset	Tsimane’	hitch	hitch	0.88	[0.31, 0.4]	51	≤0.001
Subset	American	sort	color_bin	0.99	[0.57, 0.71]	5	≤0.001
Subset	American	sort	num_bin	0.80	[0.33, 0.64]	5	≤0.001
Subset	American	sort	col_num	0.80	[0.36, 0.65]	5	≤0.001
Subset	American	double	color_bin	1.00	[0.62, 0.73]	5	≤0.001
Subset	American	double	num_bin	0.90	[0.64, 0.89]	5	0.031
Subset	American	double	col_num	0.90	[0.64, 0.92]	5	0.086
Subset	American	double	num_alt	0.50	[0.12, 0.47]	5	0.009
Subset	American	hitch	color_bin	0.98	[0.65, 0.84]	5	≤0.001
Subset	American	hitch	num_bin	0.53	[0.15, 0.47]	5	0.002
Subset	American	hitch	col_num	1.00	[0.76, 0.96]	5	0.005
Subset	American	hitch	hitch	0.73	[0.19, 0.53]	5	≤0.001
CP	Tsimane’	sort	color_bin	0.98	[0.58, 0.64]	21	≤0.001
CP	Tsimane’	sort	num_bin	0.72	[0.39, 0.53]	21	≤0.001
CP	Tsimane’	sort	col_num	0.74	[0.43, 0.57]	21	≤0.001
CP	Tsimane’	double	color_bin	1.00	[0.65, 0.68]	21	≤0.001
CP	Tsimane’	double	num_bin	0.98	[0.65, 0.78]	21	≤0.001
CP	Tsimane’	double	col_num	0.98	[0.69, 0.81]	21	≤0.001
CP	Tsimane’	double	num_alt	0.55	[0.16, 0.31]	21	≤0.001
CP	Tsimane’	hitch	color_bin	0.97	[0.65, 0.72]	21	≤0.001
CP	Tsimane’	hitch	num_bin	0.90	[0.22, 0.35]	21	≤0.001
CP	Tsimane’	hitch	col_num	1.00	[0.8, 0.89]	21	≤0.001
CP	Tsimane’	hitch	hitch	0.90	[0.28, 0.41]	21	≤0.001
CP	American	sort	color_bin	0.96	[0.66, 0.73]	18	≤0.001
CP	American	sort	num_bin	0.72	[0.39, 0.55]	18	≤0.001
CP	American	sort	col_num	0.76	[0.41, 0.58]	18	≤0.001
CP	American	double	color_bin	0.98	[0.65, 0.73]	19	≤0.001
CP	American	double	num_bin	0.76	[0.65, 0.8]	19	0.177
CP	American	double	col_num	0.82	[0.68, 0.83]	19	0.065
CP	American	double	num_alt	0.53	[0.16, 0.36]	19	≤0.001
CP	American	hitch	color_bin	0.87	[0.68, 0.77]	19	≤0.001
CP	American	hitch	num_bin	0.62	[0.18, 0.36]	19	≤0.001
CP	American	hitch	col_num	0.97	[0.82, 0.91]	19	≤0.001
CP	American	hitch	hitch	0.61	[0.24, 0.42]	19	≤0.001

Table S3: **Full accuracy results from Experiment 2**, where accuracy is measured as edit distance to nearest correct pattern (as proportion of its length). Observed means were compared to means computed over 10,000 permutations.

CP	Condition	Algorithm	Mean	95% CI	N	P
Subset	Extend	GBBG	0.39	[0.29, 0.34]	19	≈1.000
Subset	Extend	GBBOOO	0.23	[0.4, 0.46]	19	≤0.001
Subset	Extend	GBGB	0.05	[0.33, 0.38]	20	≤0.001
Subset	Extend	GBOOBG	0.29	[0.4, 0.45]	19	≤0.001
Subset	Extend	GGGBBB	0.09	[0.31, 0.37]	18	≤0.001
Subset	Extend	GOBO	0.29	[0.46, 0.51]	18	≤0.001
Subset	Integrate	GBBG	0.37	[0.5, 0.6]	17	≤0.001
Subset	Integrate	GBBOOO	0.37	[0.58, 0.65]	13	≤0.001
Subset	Integrate	GBGB	0.20	[0.47, 0.56]	19	≤0.001
Subset	Integrate	GBOOBG	0.44	[0.59, 0.67]	15	≤0.001
Subset	Integrate	GGGBBB	0.31	[0.51, 0.6]	11	≤0.001
Subset	Integrate	GOBO	0.36	[0.56, 0.67]	16	≤0.001
Subset	Translate	GBBG	0.17	[0.38, 0.52]	18	≤0.001
Subset	Translate	GBBOOO	0.10	[0.52, 0.64]	15	≤0.001
Subset	Translate	GBGB	0.03	[0.36, 0.47]	19	≤0.001
Subset	Translate	GBOOBG	0.19	[0.5, 0.6]	15	≤0.001
Subset	Translate	GGGBBB	0.13	[0.43, 0.57]	14	≤0.001
Subset	Translate	GOBO	0.16	[0.47, 0.58]	17	≤0.001
CP	Extend	GBBG	0.37	[0.29, 0.34]	23	≈1.000
CP	Extend	GBBOOO	0.11	[0.4, 0.46]	24	≤0.001
CP	Extend	GBGB	0.03	[0.33, 0.38]	23	≤0.001
CP	Extend	GBOOBG	0.23	[0.4, 0.45]	24	≤0.001
CP	Extend	GGGBBB	0.05	[0.31, 0.37]	24	≤0.001
CP	Extend	GOBO	0.23	[0.46, 0.51]	23	≤0.001
CP	Integrate	GBBG	0.43	[0.5, 0.6]	22	≤0.001
CP	Integrate	GBBOOO	0.30	[0.58, 0.65]	20	≤0.001
CP	Integrate	GBGB	0.21	[0.47, 0.56]	20	≤0.001
CP	Integrate	GBOOBG	0.45	[0.59, 0.67]	21	≤0.001
CP	Integrate	GGGBBB	0.24	[0.51, 0.6]	22	≤0.001
CP	Integrate	GOBO	0.29	[0.56, 0.67]	19	≤0.001
CP	Translate	GBBG	0.07	[0.38, 0.52]	23	≤0.001
CP	Translate	GBBOOO	0.04	[0.52, 0.64]	20	≤0.001
CP	Translate	GBGB	0.00	[0.36, 0.47]	24	≤0.001
CP	Translate	GBOOBG	0.15	[0.5, 0.6]	23	≤0.001
CP	Translate	GGGBBB	0.02	[0.43, 0.57]	22	≤0.001
CP	Translate	GOBO	0.05	[0.47, 0.58]	23	≤0.001
