# Supplementary material for: Children use algorithm induction to discover patterns in data
Source: Nat Commun. 2026 May 30;17:7017. doi: 10.1038/s41467-026-73029-9 (PMC13392036; doi:10.1038/s41467-026-73029-9)
Supplement: Supplementary file 2 — Reporting Summary [file 41467_2026_73029_MOESM2_ESM.pdf]

nature portfolio

Corresponding author(s): Benjamin Pitt

Last updated by author(s): 02/03/2026

Reporting Summary

Nature Portfolio wishes to improve the reproducibility of the work that we publish. This form provides structure for consistency and transparency in reporting. For further information on Nature Portfolio policies, see our Editorial Policies and the Editorial Policy Checklist.

Statistics

For all statistical analyses, confirm that the following items are present in the figure legend, table legend, main text, or Methods section.

n/a	Confirmed	
☐	☒	The exact sample size (*n*) for each experimental group/condition, given as a discrete number and unit of measurement
☐	☒	A statement on whether measurements were taken from distinct samples or whether the same sample was measured repeatedly
☐	☒	The statistical test(s) used AND whether they are one- or two-sided<br>*Only common tests should be described solely by name; describe more complex techniques in the Methods section.*
☐	☒	A description of all covariates tested
☒	☐	A description of any assumptions or corrections, such as tests of normality and adjustment for multiple comparisons
☐	☒	A full description of the statistical parameters including central tendency (e.g. means) or other basic estimates (e.g. regression coefficient) AND variation (e.g. standard deviation) or associated estimates of uncertainty (e.g. confidence intervals)
☐	☒	For null hypothesis testing, the test statistic (e.g. *F*, *t*, *r*) with confidence intervals, effect sizes, degrees of freedom and *P* value noted<br>*Give P values as exact values whenever suitable.*
☐	☒	For Bayesian analysis, information on the choice of priors and Markov chain Monte Carlo settings
☐	☒	For hierarchical and complex designs, identification of the appropriate level for tests and full reporting of outcomes
☐	☒	Estimates of effect sizes (e.g. Cohen's *d*, Pearson's *r*), indicating how they were calculated

Our web collection on statistics for biologists contains articles on many of the points above.

Software and code

Policy information about availability of computer code

Data collection	No software was used to collect the data.
Data analysis	Models are described in the Methods section. All data and analysis scripts are freely available online at osf.io/eawkh/.

For manuscripts utilizing custom algorithms or software that are central to the research but not yet described in published literature, software must be made available to editors and reviewers. We strongly encourage code deposition in a community repository (e.g. GitHub). See the Nature Portfolio guidelines for submitting code & software for further information.

Data

Policy information about availability of data

All manuscripts must include a data availability statement. This statement should provide the following information, where applicable:

- Accession codes, unique identifiers, or web links for publicly available datasets
- A description of any restrictions on data availability
- For clinical datasets or third party data, please ensure that the statement adheres to our policy

All data and analysis scripts are freely available online at osf.io/eawkh/.

Research involving human participants, their data, or biological material

Policy information about studies with human participants or human data. See also policy information about sex, gender (identity/presentation), and sexual orientation and race, ethnicity and racism.

Reporting on sex and gender	The study was designed without consideration of participant's sex or gender, and the results are not specific to any one group. Participants' sex was noted by researchers and not analyzed.
Reporting on race, ethnicity, or other socially relevant groupings	Our study included Tsimane' and US American children. We identified Tsimane' children on the basis of their location at testing (i.e. Tsimane' villages in rural Bolivia), parental identity (i.e. Tsimane'), and native language (i.e. Tsimane'). We identified US children on the basis of their location at testing (i.e. preschools in the San Francisco Bay Area), and spoken language (i.e. English).
Population characteristics	See below
Recruitment	Tsimane' participants were recruited in their villages. The opportunity to participate in our study was announced over the radio in advance of our arrival and then again by voice the researchers upon arrival. US participants were recruited in their classrooms in Berkeley, CA. All eligible participants were invited to participate. The most likely self-selection bias, if any, may be an overrepresentation of scientifically, culturally, or socially curious children and the potential effects of such a bias are not clear.
Ethics oversight	Institutional Review Board at the University of California, Berkeley and the El Gran Consejo Tsimane' (The Tsimane' Grand Council)

Note that full information on the approval of the study protocol must also be provided in the manuscript.

Field-specific reporting

Please select the one below that is the best fit for your research. If you are not sure, read the appropriate sections before making your selection.

☐ Life sciences ☒ Behavioural & social sciences ☐ Ecological, evolutionary & environmental sciences

For a reference copy of the document with all sections, see nature.com/documents/nr-reporting-summary-flat.pdf

Behavioural & social sciences study design

All studies must disclose on these points even when the disclosure is negative.

Study description	Quantitative study
Research sample	Our research sample includes indigenous Tsimane' and US American children. We chose to test Tsimane' children because they live in a culture with less emphasis on exact counting and numbers, where subset-knowers are found among older children. Studying two distinct cultures also allows us to test the generalizability of the findings. Study 1 tested 72 Tsimane' children (30 female; ages 3 - 13 years; mean age = 7.5 years, mean schooling = 1.4 years) and 24 US children (14 female; ages 3:1 - 5:5; mean age = 4.4 years, mean schooling = 1.4 years). Study 2 tested 43 (21 female; age range: 7-12 years; mean age = 9.3 years, mean schooling = 3.6 years). These samples were not designed to be representative of the larger populations.
Sampling strategy	Because our study asks about basic cognitive processes that may be found in any group, we used convenience sampling in each of our populations. We set target sample sizes for each experiment; Final sample sizes were determined by the availability of researcher time (e.g. in the field) and willing participants (e.g. in a classroom).
Data collection	All data was behavioral and was recorded on paper by a trained researcher and most testing sessions were video and audio recorded. A second research was also sometimes present to observe and/or translate. All variables were within-subject so there was no between-subject experimental conditions to which experimenters might be blind. Participant's parent was sometimes present in the room during testing, but was instructed not to interfere (and any interference or help was noted). Participants were children and therefore we collected informed consent from their parent or guardian and assent from them. Parents and children were compensated with small gifts, regardless of their performance or completion. All raw data and analysis scripts are publicly available online at https://doi.org/10.17605/OSF.IO/EAWKH.
Timing	For Experiment 1, US data was collected during a series of brief testing sessions at preschools during the 2019-20 academic year. Tsimane' data was collected during a two field trips to Bolivia during two field trips: 5/28/2019 to 6/8/2019 and from 8/20/2019 to 9/25/2019. For Experiment 2, data was collected between 7/8/2022 and 6/9/2022.
Data exclusions	No participants were excluded from analysis. A small portion of participants' data was excluded when they (a) failed to follow instructions or (b) failed a simple comprehension check for a given task, as specified in the manuscript.
Non-participation	Our recruitment strategy included many eligible people (in classrooms and in the field), and only some of them participated. We cannot say what number of eligible people declined to participate when given the opportunity. To our knowledge, no participants dropped out of testing.

Randomization | Participants were not assigned to experimental groups.

Reporting for specific materials, systems and methods

We require information from authors about some types of materials, experimental systems and methods used in many studies. Here, indicate whether each material, system or method listed is relevant to your study. If you are not sure if a list item applies to your research, read the appropriate section before selecting a response.

Materials & experimental systems

n/a	Involved in the study
☒	☐ Antibodies
☒	☐ Eukaryotic cell lines
☒	☐ Palaeontology and archaeology
☒	☐ Animals and other organisms
☒	☐ Clinical data
☒	☐ Dual use research of concern
☒	☐ Plants

Methods

n/a	Involved in the study
☒	☐ ChIP-seq
☒	☐ Flow cytometry
☒	☐ MRI-based neuroimaging

Plants

Seed stocks	NA
Novel plant genotypes	NA
Authentication	NA
