# Supplementary material for: Children use algorithm induction to discover patterns in data
Source: Nat Commun. 2026 May 30;17:7017. doi: 10.1038/s41467-026-73029-9 (PMC13392036; doi:10.1038/s41467-026-73029-9)
Supplement: Supplementary file 3 — Transparent Peer Review file [file 41467_2026_73029_MOESM3_ESM.pdf]

nature portfolio

# Peer Review File

# Children use algorithm induction to discover patterns in data

Corresponding Author: Dr Benjamin Pitt

**This file contains all reviewer reports in order by version, followed by all author rebuttals in order by version.**

Version 0:

Reviewer comments:

Reviewer #1

(Remarks to the Author)
This paper raises a very interesting issue – can uneducated children infer the logic or "program" behind a visuo-motor pattern? Furthermore, the experiments are clever and innovative, especially the second one where participants have to extend patterns in various ways, and some degree of generalization is observed. Unfortunately, however, as I argue below, both the design and the analyses suffer from problems which imply that the results do not (yet?) support the very strong conclusions that are proposed. Below, I list a number of problems.

Design. The program inference hypothesis that the authors propose does not seem to be very directly supported by the data. Rather, the authors show that it provides a better fit than the three n-gram alternatives that they propose. I am a bit worried about this approach for several reasons:
1. Do they consider enough alternative models? There is a huge gap between ngram models and the very sophisticated programing language that they proposed. According to Dehaene et al's (2015) hierarchy of sequence knowledge, intermediate levels could involve numerical knowledge and chunking (for instance the children might learn that "blue black" is one chunk and "red black" is another chunk). It seems to me that, at least for experiment 1, such knowledge could suffice to respond accurately and explain the observed behavior. It would be great if the authors could reject those alternative models, but at the very least they should be discussed.
2. Even within the low-level models of transition probabilities, the n-gram models tested by the authors may not be the most appropriate. A new idea is the free-energy model or "thick past", where the idea is that the (temporally discounted) average of recent items is used as a predictor of the next one. This is similar to a 1-gram model, but no longer solely based on the most recent item. Again, I think that this view (or related skip-gram models) could easily account for the data.
3. N-grams for n>1 are notoriously difficult to estimate, and here it seems that this is the worst possible situation because the data is extraordinarily limited. Given the difficulty of estimating a 2-gram or 3-gram model from such scarce data, it should not be surprising that the model fit is so poor. At the very least, details of how those n-gram models are implemented would be appreciated.

Statistics. On line 272: what do you mean by "we permuted participants' responses". Did you permute the labels, i.e. the pairings of response patterns to the 3 stimulus patterns? Or did you shuffle every single response within a pattern? The second choice is inappropriate, as it may overestimate the chance level (if, for instance, participants spontaneously tend to alternate their responses regardless of the proposed pattern; what must be shown is that they produce distinct patterns in different conditions). The massive significance of the results, in the face of what are, in fact, extremely irregular responses (figure 1), suggests to me that you may have adopted the second approach, which is inappropriate in my opinion. It is less clear to me whether this problem also affects experiment 2, but it might.

Other minor comments:
- Line 232: in experiment 1, were the three patterns presented in fixed or in random order? If the latter, can you exclude that the later responses include a significant component of "meta-learning" about the types of patterns allowable in the task? See eg. Participants 207 or 244 who are, surprisingly, performing better on the most complex pattern (Hitch).
- Line 236. It took me several readings to understand what was meant by the Hitch description (I initially thought that the black ball, conventionally, depicted either the blue or the red). Perhaps this sentence could be improved: "we sorted one pair of colors while including a third color at each step"

(Remarks on code availability)

Reviewer #2

(Remarks to the Author)
Overview: This is a wonderful paper, and will be an important addition to a wide range of literature in cognitive science. The topic is of broad enough interest to merit publication in Nature Communications, the conclusions is more than interesting enough, and the methods are robust.

I often give pages and pages of referee comments, but have very few comments here as I found the paper to be edifying, clear, and excellent all around. Here are the questions I would most like the authors to answer.

1) Although it seems like a side comment, the claim about Tsimane not having standardized words for geometric shapes seems hard to believe. (The authors write “Many Tsimane’ have little or no formal schooling, do not have standardized words for basic geometric shapes (like circle, square, and triangle), and many adults do not read, write, or use math.” There is a Butterworth et al. citation on the sentence before this claim—is that what is supposed to substantiate the standardized word claim? I know this seems like a small point but part of why I think the paper should be accepted is that it is just very rigorous and believable (which is hard in field work and often not quite true of papers in Science, PNAS). So I would like a little more substantiation of this claim, as it was the only one in the paper that I found to be quite unintuitive.

2) Is there a difference between program induction and learning a rule in the language of thought? The more I think about it, the less daylight there seems to be here. Some discussion on what the differences are, or how to pull it apart, could be edifying.

3) Why think the second part of this sentence? “This pattern of results suggest that children across cultures and ages can infer the structure of novel patterns even before they have learned to count, and that this understanding does not reflect a single moment of insight, but rather a piecemeal process of inferring the individual algorithmic features of the input.” It goes against so much of learning, which is often not stepwise (not just one-shot word learning either; see, e.g., the Gallistel and King 2009 book).

4) I’m also wondering how much of the anti-nativist implicature here is on purpose. Why are these results inconsistent with nativism (which isn’t said, but is implied)? Is it that they are the output of a domain-general learning mechanism? I would’ve thought the evidence here was equally suggestive of a set of prelinguistic, universally shared conceptual primitives. At the very least the paper is fairly anti-Whorfian, and it could be worth noting that (at least for theorists like Gary Lupyan who think that Whorf is back, and true). Maybe I am misreading and the implicature isn't really there? Perhaps it would be best to include a short section or paragraph on how you think these results interact with debates about nativism.

Also in the Introduction, you are missing a ‘to’ in the second sentence (062)

But these claims shouldn't detract from the main point: its an excellent paper, full of difficult to gather data.

All best,

Eric Mandelbaum
(I always sign my reviews)

(Remarks on code availability)

Reviewer #3

(Remarks to the Author)
The authors present an intriguing argument for and novel (as far as I'm aware) data bearing on the possibility of domain-general program induction as a human-universal cognitive mechanism which underpins human-unique learning abilities.

The methodology and analysis of this work seems solid and I particularly commend the effort to work with atypical, hard-to-reach populations.

However, it seems to me that several gaps remain within the current manuscript - both in its overall logic and in the connection between the evidence presented and the conclusions the authors wish to draw:

1 - The notion that program induction may support human-unique learning is emphasized strongly in both introduction and conclusion of the piece. At the same time I see no reference to evidence that non-human animals are unable to complete the kind of inductive tasks presented here. I suggest that the authors either provide such evidence, or drop the claim that the mechanisms they are studying are necessarily human-unique. The point is hardly trivial - after there is an extensive literature showing that non-human animals struggle with, but are ultimately capable of, abstract relational reasoning (e.g. relational matching).

2 - Is it not possible that the kind of patterns presented here could be processed by an initially domain specific mechanism? I

have in mind the ability of children to flexibly learn language across any modality (sight, sound, touch). Given that language-learning requires much more complex program induction than the present work, in what way can we distinguish the present results from yet another expression of the remarkable human ability to learn systems of symbolic communication? The task is, after all, quite clearly a communicative context.
For sake of illustration, if we presented schooled, Western individuals with a task in a repetitive, unfamiliar language and asked them to extend the patterns therein - would we not also find the kind of noisy-but-program-like responses reported here?

3 - The analyses, as they are presented, do not adequately control for the effects of formal schooling. Specifically, while the authors report data from children with no school experience in Experiment 1, no such data are reported for Experiment 2. This is a crucial point given that the pattern-following in Experiment 2 is, by the authors own admission, crucial for attributing program-induction. My concern is that decades of evidence in cross-cultural psychology has shown that exposure to even minimal amounts (e.g. one year) of schooling can dramatically change patterns of response on cognitive tasks, including those involving abstract patterns (see e.g. Luria, 1976; Sharp et al., 1979). Without data from unschooled participants in Experiment 2, then, it is difficult to be confident in the authors assertion that "Presented with small amounts of data, children across cultures can induce the abstract logical structure therein, constructing ad-hoc explanatory theories without... formal schooling."

4 - I have a hard time interpreting Fig 4: In commonsense terms, what does it mean for participants to be 'above chance' in replicating the patterns?
Also, my suspicion is that chance is a very poor baseline here. After all, I'd bet that most humans, when given a series of geometric shapes to arrange, will make some kind of pattern out of them (given that all the cultural groups I can think of have some kind of geometric decorative motifs, more on which below).
If this is the case, it is possible that 'above chance' performance is driven in whole or part by individuals just arranging the shapes into patterns of their own choosing. This would also drive the 'program-like' result displayed in 4b.
Of course I may be missing a reason why such an interpretation is untenable - but if so I would appreciate a brief discussion by the authors.

5 - Relatedly, the authors emphasize that the task children are presented with is completely unfamiliar to them. But in what sense are they new? And how do we know this?
It is obviously the case that the specific set of stimuli and instructions will be new - as they would be to any participant who had not completed this specific task. Consequently the 'novelty' must be at the level of 'this type of task'. But why should we assume that Tsimane children are unfamiliar with this kind of task?
For instance, (unless I'm misremembering) the Tsimane use geometric patterns for face painting - in many cases using the very same shapes and colors (e.g. red squares, black circles) as the tasks presented here. Let us assume (I think plausibly) that the patterns each individual paints are neither exact repetitions of previous instances, nor totally random. From this it follows that what individuals have learned is the ability to replicate some abstract scheme or aesthetic. Is this not familiarity with something like the present task? If not, why not?
More generally, what is the type of task used here such that we can be confident that Tsimane participants will not be familiar with it?

In sum, the methodology and findings of the present work are intriguing. The ambitious scope of the argument is also a good match for Nature Communications (e.g. "program induction may be central to both our cognitive flexibility and cultural diversity, allowing people to discover and generalize the latent algorithmic structure of their natural and cultural environment, whatever it may be").
That being said, I am not yet convinced by either the logic of the authors' claims, or by the way the data they present supports these claims. I suggest that a substantial revision is in order for the manuscript to be accepted.

Minor notes:
- It would be useful for the authors to discuss how exactly they translated their instructions to recreate 'the same pattern'. In other small-scale languages I am aware of there is no easy translation of this term.
- Figure 3a is crucial to illustrating the program-induction argued to drive the present results. Yet, the key element of this figure is a "modal response" from Tsimane participants. This feels needlessly obscure to me - leaving open the possibility that e.g. the modal pattern was repeated twice - while all other responses were unrelated to the presented pattern and just abided by some pattern of their own (which would drive the Fig 4b data). It would be extremely helpful to have, in supplemental information, the full set of responses for both Experiment 1 and 2 displayed in the style of Fig 1.

(Remarks on code availability)

Reviewer #4

(Remarks to the Author)
The manuscript entitled “Algorithm induction in indigenous Amazonian children” explores the cognitive mechanisms of learning using a program induction framework. Thanks to two experiments conducted among children coming from an

Indigenous community (the Tsimane´ in Bolivia) and from the USA, the authors bring evidence that children can infer patterns early in life and may learn novel patterns more effectively through induction than imitation or substitution. Supported by rigorous methodologies and a rich dataset, this work has the potential to make a meaningful contribution to the field of cognitive development, particularly in understanding how children learn across diverse cultural contexts.By the innovative aspect of their work and the implication for our understanding of human cognitive development, there is no doubt that this work is a relevant contribution to the field and meaningful for Nature Communication journal.
While the study is insightful, several critical concerns must be addressed before it can be considered for publication. Three main aspects should be specifically addressed: 1) the lack of a rigorous ethical demarch; 2) the justification of working with north American children; and 3) the extent of the interpretation. Additionally, the manuscript's accessibility to non-specialists could be improved by reducing jargon and expanding explanations. Below, I have detailed my main concerns, as well as minor comments and suggestions, that, I hope, will help the authors to review this work.

Main concerns.
1) Lack of a rigorous ethical demarch.
My main concern relates to the ethical approach taken by the authors and encompasses several aspects of the study.
Ethical Approval Documentation
First, the ethical approval obtained from institutional and local organizations should be explicitly stated, including their approval numbers. Was ethical approval obtained from the Bolivian National Ministry of Science? If not, this omission should be clearly justified.
Informed Consent Procedures
In addition to institutional ethical approval, the authors should have obtained Free, Prior, and Informed Consent (FPIC) from every individual involved in the study. Currently, the manuscript does not clearly specify whether or how informants' consent was obtained. The authors state that they "received assent from all participants and consent from their legal guardians," but further details are necessary. How was the research presented to the children? What measures were taken to ensure that participants fully understood the study's aims, methods, risks, benefits, and data usage? How was children's consent obtained?
Clearly articulating the ethical procedures is essential for any research involving human participants, particularly when working with Indigenous Peoples and children. Research with children requires adherence to specific guidelines on presenting the research and obtaining consent. For example, see Graham, A., Powell, M. A., & Taylor, N. (2015). Ethical research involving children: Encouraging reflexive engagement in research with children and young people. Children & Society, 29(5), 331–343.
Researcher Positionality
The positionality of the authors who conducted the data collection should be discussed. Potential biases and limitations arising from differences in age, culture, or gender between the researchers and the children involved in the study should be explicitly addressed.
Contextual Embedding
The description of the Tsimane' community is overly brief and outdated. The study should be better contextualized within the realities of the communities involved. Currently, the manuscript relies on a single, outdated reference to describe the population, reducing the description of the Tsimane' to approximately six lines. This brevity not only creates a gap in the manuscript but also poses challenges for interpreting the findings within the appropriate cultural context. A more comprehensive account, supported by recent literature, would provide a richer and more accurate foundation for interpreting the results.

2) The inclusion of North American children.
While the authors present and use data from two different settings, they do not clearly articulate the comparative aspect of this research, nor do they fully leverage the comparison to better interpret their findings. For instance, although US children appeared to have lower accuracy scores than Tsimane' children in Experiment 1, the authors did not discuss this difference. The data from US children is limited to Experiment 1 (see lines 822–830), and the sample size is three times smaller than that of the Tsimane' group. Furthermore, the individual characteristics of US children are not thoroughly considered but rather assumed. Factors such as schooling, familial, social, and cultural contexts, as well as health, may significantly affect cognitive development. For the Tsimane', schooling is assumed to be low, but how was this assessed? What kind of data did the authors use as a proxy for schooling?
Given the reduced sample size and the data being restricted to Experiment 1, is it sufficient to support generalizations? This study could undoubtedly provide strong evidence of cognitive mechanisms related to human learning processes without including the US sample. Such a focus might also encourage further research in other cultural and social settings. Simplifying the analysis to include only the Tsimane' data would better highlight the study's key insights and improve clarity. Therefore, I question the relevance of including data from US children. What does it contribute to the overall conclusions of the work? The comparative aspect of this study should be better justified, clearly presented in the results, and fully interpreted in the discussion. Alternatively, the authors might consider focusing solely on the Tsimane' sample, which would allow them to situate their study more effectively within the cultural and social-ecological context of the children they are studying. This would also simplify the manuscript, which is currently dense and complex.
3) The extent of the interpretations.
The discussion section is concise but lacks depth and broader contextualization. The discussion might be reviewed to better structure it around clear insights and to extent the insights provided by this study not only for the field of cognitive development at the theoretical or methodological insights, but also regarding what these insights bring to human development in general, or to Tsimane´ learning process and livelihood. Also, if the comparative aspect of the study is maintained, it should be further discussed.

Finally, the language used in the manuscript might be improved to avoid employing too often specialized “jargon” and to rather try to open the work to a broader academic audience.

Minor comments:

Introduction
Clarify ambiguous phrases:
- “people learn so much from so little” Specify what “so little” refers to.
- “basic domains of knowledge” provide examples to clarify it
- Why assuming observation is “simple”?
- Please explain what “improvisation intelligence” refers to

Paragraph from Line 115 to 141: Adding age range to the statements formulated would help to situate the insights brought. For instance, specific what is understood here by prelinguistic infants (what is the age range)? What children and infants mean here (both categories might largely differ depending on the cultural and social contexts)?
Most importantly: the description of the Tsimane´ should be much more developed and nourished by updated references. A large amount of research has been conducted among the Tsimane´, including studies on memory, learning process, and schooling. Please update this information and expand this paragraph to better situate the study within local realities.
Consult the work made by Gurven and colleagues, and for instance the following ones:
Schniter et al., 2015 Skill ontogeny among Tsimane forager-horticulturalists
https://onlinelibrary.wiley.com/doi/abs/10.1002/ajpa.22757
Reyes-García et al 2016 Schooling, Local Knowledge and Working Memory: A Study among Three Contemporary Hunter-Gatherer Societies https://journals.plos.org/plosone/article?id=10.1371/journal.pone.0145265#pone.0145265.ref045
Gurven et al., 2017 Cognitive performance across the life course of Bolivian forager-farmers with limited schooling.
https://psycnet.apa.org/record/2016-41992-001

Results
Some aspects of the results might be moved to the Discussion part in order to gain in clarity. Consider moving interpretive statements, as for instance the sentences in Line 292-301 that might rather go to the discussion.
If the authors prefer keeping the interpretations within the Results part, they might consider restructuring their manuscript by having a section of “Interpreted Results” and then only a “Conclusion” section (without a discussion part).

Discussion
The discussion is quite short and might be improved by being restructured around two or three main aspects, and further developing the implications of this research insights in each one of these. Now, the discussion lacks a broader perspective and would gain by not only stating this study work within cognitive development in general, but maybe, also, in what these insights might tell us about Indigenous learning process, by further developing on the cross cultural potential aspects, etc.
Add reference for the sentence finishing line 561.

Methods:
- Specify who conducted the data collection and provide their qualifications or roles.
- Include the age range of participants in both experiments (not just average ages).
- Clarify the timeline of Experiment 1.

References:
A lot of typographical errors remain in the manuscript. For instance, the authors should change their “???” by actual data. This is the case line 966, line 1018, 1027, 1032, 1086, 1101,1251, 1311, 1317, 1351, 1358, and 1360
Line 993: “theory” is repeated. Delete one

(Remarks on code availability)

Version 1:

Reviewer comments:

Reviewer #2

(Remarks to the Author)
After revisions I am a bit more confused by the author's overall theoretical outlook (particularly why they take this to be consistent, never mind supportive of Whorf) but I don't think that is a reason to hold up publication of this very interesting and accessible work. I think it is acceptable as is.

(Remarks on code availability)

Reviewer #3

(Remarks to the Author)
Responding to the points in order

1 - The revision addresses my concern, thank you.

2 - I take the point that 'language learning' applied to a sufficiently broad set of domains becomes de facto domain general. However the authors argument that the task was somehow non-communicative makes no sense to me: They admit that "the context of any experiment is in some way communicative" but then say "our tasks were not." I agree with the first statement, and don't see how the second can possibly follow. Nor do I see how the nature of the patterns or lack of feedback make the task non-communicative. The experimenter has asked the participant to do something, and the participant will (correctly) assume that the experimenter will check and interpret their performance in some way. I fail to see how this is a non-communicative context — or at the very least how the authors can rule out that participants interpret it as one.
I'm also confused by the asymmetry in the new text provided by the authors: They point out that domain-specific mechanisms may support performance here, but are not necessary to explain it. But is the same not true of domain-general mechanisms? Given the communicative nature of the context, and the obvious potential of patterns to act as symbolic language, can we not just as well say that performance could be supported by domain-general mechanisms, but that these are not necessary to explain it? I feel like the broader point may be that we don't have sufficient conceptual clarity on the difference between 'truly domain general' and 'broad but domain-specific' capacities. If this is the case, then it feels like the heavy emphasis specifically on domain generality should be softened (e.g. to 'broad, potentially wholly domain-general capacities')

3 - I don't see how the additional paragraph addresses the concern about formal schooling driving the observed effects. As I mention in the original review, even very limited amounts of formal schooling have been shown to produce qualitative differences in performance (see Luria's work, Cole et a., 1971; Sharp et al., 1979 etc.) The difference need not be in the learning of new representational systems (e.g. number) — it is plausible that simply learning to look for the kind of 'correct' or 'perfect match' solution within decontextualized tasks emerges as a kind of activity from participating in school.
The paragraph added by the authors does not address this concern, nor does it acknowledge the fact that the main effect seems to be confounded with schooling exposure. Instead it appears to gloss over this issue, gesturing towards the fact that a wide variery of experiences may 'sharpen program-induction abilities.' Surely this begs the question by presupposing the very program-induction abilities the authors claim to be demonstrating?
I see this as a crucial point to address in the final manuscript — even if only to say that future work will have to disentangle the present results from the effects of formal schooling. (Though of course I remain open to new arguments why this may not relevant after all).
Note, as I will mention below, I don't actually think that schooling can produce program-induction, or any other new 'fundamental' capacity. My point, rather, is that schooling has been overwhelmingly shown to impact performance on decontextualized tasks with arbitrary content, and thus should be seriously considered as a factor in the pattern of data presented here.

4 - An excellent clarification, thank you.

5 - First, a minor point on stimulus familiarity: Inhibition of relational reasoning by superficial similarity has been shown specifically when superficial matches compete with relational ones. Simply being familiar with the materials has not been shown to inhibit relational reasoning with them — as far as I am aware. Nor do I understand why it would do so (e.g. would a mathematician very familiar with numbers be less likely to treat them relationally? Or looking through a few hundred Raven's matrices stimuli inhibit performance on the test?) That being said, the authors' arguments don't seem to rest on this point, so I am happy to leave it be. My more pressing, and general, concern is the following:

In no way do I wish to question Tsimane children's abilities to match abstract patterns — I have no doubt they are able to do so. My broader concern is that this point is an obvious one, given e.g. children's obvious ability to learn any number oflanguages and cultural patterns. In other words, what is the current study telling us that observing the wide variability in languages and pattern-making across the world's cultures does not? Or, perhaps more specifically, the fact that children rapidly assimilate to new languages and cultures when travelling or being adopted?
After all, when faced with a wholly new culture the adopted child learns tasks which are just as novel and arbitrary as the task used here. If this was somehow impossible the relevant literatures would presumably have been full of reports and attempted solutions had this been a major barrier for their development and adaptations. Instead, my impression of such work is of consistent evidence that children rapidly and flexibly adapt to even extremely variable cultural contexts.
This is not to say the present work is not saying something new. Rather, I think the contribution lies in providing a rigorous demonstration of some of these apparent capacities. To the extent that is the case the more appropriate framing, I would argue, is something like:
"Clearly humans across the lifespan are able to learn and adapt to arbitrary patterns and tasks, as evidenced by rapid cultural learning within and and across cultural contexts. Here we provide a quantitative measure of this kind of learning, applying rigorous formal criteria to the phenomena."
While this softens the impact somewhat, I happen to believe that formalizing these kinds of cross-cultural observations is very much a worthwhile goal.

In summary, I support the publication of this piece, but strongly suggest the addition of three caveats
1) there is much work to be done teasing apart 'truly domain-general' v. 'domain-specific but broad' mechanisms, and including perhaps some conceptual clarification
2) formal schooling remains to be fully controlled for in future work
3) the general capacity for learning patterns is already evident from cultural observation, and the contribution of this work is

to formalize this observation

(Remarks on code availability)

Reviewer #5

(Remarks to the Author)
Dear Authors,

I am not one of the original reviewers on this paper. Given that, I am applying the same policy that I prefer applied to my own work when new reviewers are brought on at the revision stage, which is: It is not my role to introduce new problems, but to go over the original paper, the reviews already in hand, the revision, and to see whether the revision addresses the issues. Obviously, if some extreme issue was missed by previous reviewers that needs to be mentioned, but other than that I think the role is more limited.

With that in mind:

Briefly, I think that: this is a great manuscript (before the revisions and certainly after them), the revision addresses the major issues raised by previous reviewers (or adequately explains why they were not addressed); it should be accepted, and will make a valuable contribution of interest to many people in the field.

====

With that in mind, I have a few small-level comments that the authors should feel free to address if they so choose, but these are all minor and do not stand in the way of acceptance/publication.

1. "[languages of thought] can be universal in their expressive power, capable of representing all possible computations — Here and elsewhere, there's very little consideration for the fact that something may be _in principle_ learnable (or computable, or representable) but that it might take eons to find the relevant program in a language that is ill suited to the task. 'Turing complete' turns out to be a very low bar, one that be achieved by the right combination of buckets and sand contraptions, and recently it was noted (jokingly) that Powerpoint is Turing-Complete. The question is often turns on whether there are relevant primitives or pre-processors of stimuli or learning mechanisms or search mechanisms that would allow the inference of a program in relevant time. I know the authors are highly aware of this fact, having worked on it themselves, and it isn't terribly important to expand on, but it may be worth remarking on the issue here and elsewhere as relevant (see another example below).

2. Related to the above, I get that the authors are not making strong claims about *which* LoT is being used by children, and it is sufficient to argue that there IS one (as opposed to other forms of learning). Still, I was highly interested as a reader who cares about this work to know a bit more which primitives are likely most relevant. Again, this raises the problem that a teeny-tiny LoT is likely able to learn all of these patterns in principle given sufficient time, but some LoTs are more naturally suited to it than others, and it is very interesting to consider whether this work is telling us anything about what the likely primitives of that LoT are (a-la other work by Piantadosi and his colleagues on concept learning).

3. To the paragraph staring in 210 and ending in 219 ('If program induction drives the acquisition of domain-specific algorithms
like the counting algorithm...), this again seems to me like a false dilemma, as though what is possible is either 'Program Learning' or 'Association Learning' and then success or failure on a given task may show that for *this* task 'Program Learning' wasn't available. It is also possible that one's LoT has certain primitives and operations and pre-processors that make some domains much easier to learn or figure out in a given amount of time compared to others.

4. How much, if any of this was pre-registered (sample sizes, analyses, models, etc)? I couldn't figure that out quickly from the repo or reporting summary pdf, and it doesn't seem in the paper itself that anything was pre-registered. That isn't a requirement, but it is helpful to know these days what was agreed on ahead of time, how sample sizes were determined, what else was tried, and so on. If this was mentioned somewhere and I missed it, my apologies.

5. "Likewise here, the inductive process was not all-or-nothing: children often inferred some of the features of the sample patterns, while missing others." - this is highly interesting and relates to (2), it left the reader wondering which features were inferred and which were missed, assuming there was any systematicity to it, it can inform the LoT being used (if at all).

7. To this bit in the discussion:

" Whereas some accounts attribute humans’ extraordinary cognitive abilities to qualitative differences in learning mechanisms [14, 53, 62], others attribute them to quantitative differences in information processing capacity [136]. In model systems, differences in memory alone can have profound impacts on what can be learned, holding constant the amount of data and the inferential mechanisms. In this way, program induction could be fundamental to learning in humans and non-human animals alike, despite dramatic differences in their cognitive abilities."

-- I get that this was in response to a reviewer comment, and also I appreciate the connection to previous work [specifically Cantlon and Piantadosi 2024], but it left things a little bit unclear here. Is the claim here that animals CAN produce these patterns? Or that they CAN'T produce these patterns, BUT that the reason that they CAN'T is not due to a lack of LoT but rather to information bottle-necks within an LoT? It seems the authors are trying to simply not get into it whether animals can or can't do this, saying something like "weeellll, maybe they can and maybe they can't but if they can't, we can explain that as [x]". I realize this is likely outside the scope of the current paper but such a claim would've led me to suggest the authors actually model that, meaning they be more specific about using something like their own LoT but under different memory limitations and showing the point at which it breaks down. That could produce novel predictions for what non-human animals pass or fail, or what humans can be expected to learn under memory-stress conditions.

8. "To be clear, these findings do not rule out domain-specific learning mechanisms" -- This goes back to points (1 and 2), but here and elsewhere in the discussion, I think a better perspective on 'domain specific' knowledge is not 'domain specific *learning mechanisms*" but rather on domain-specific primitives, algorithms, and representations. Imagine you had a program-induction model that had as primitives stuff like "Object" and "Near" and "Rotated" and pre-processing that takes in pixels and produces 3D object representations. Obviously such a model would have an easier time learning stuff to do with space and physics, as opposed to other patterns outside of that domain, but it isn't because it has a different *learning mechanism*, the learning mechanism might be the same: Bayesian induction over programs via sampled search. I want to emphasize I am *not* saying that that is the learning mechanism, nor am I saying there is only one learning mechanism, I am saying it seems at least possible (and more in line with previous developmental work) to distinguish domain-specific data and patterns as being about primitives than 'learning-mechanisms'.

(Remarks on code availability)

Version 2:

Reviewer comments:

Reviewer #3

(Remarks to the Author)
The authors have satisfactorily addressed all of my concerns, I am happy to recommend the paper for publication.

(Remarks on code availability)

Reviewer #5

(Remarks to the Author)
I thought the paper was already acceptable-for-publication in my previous review, and I appreciate the authors addressing my optional additional comments in that review, it seems all good to me, and I look forward to seeing it officially out.

(Remarks on code availability)

**Open Access** This Peer Review File is licensed under a Creative Commons Attribution 4.0 International License, which permits use, sharing, adaptation, distribution and reproduction in any medium or format, as long as you give appropriate credit to the original author(s) and the source, provide a link to the Creative Commons license, and indicate if changes were made.
In cases where reviewers are anonymous, credit should be given to 'Anonymous Referee' and the source.
The images or other third party material in this Peer Review File are included in the article's Creative Commons license, unless indicated otherwise in a credit line to the material. If material is not included in the article's Creative Commons license and your intended use is not permitted by statutory regulation or exceeds the permitted use, you will need to obtain permission directly from the copyright holder.
To view a copy of this license, visit https://creativecommons.org/licenses/by/4.0/

**REVIEWER COMMENTS**

**Reviewer 1** ################

This paper raises a very interesting issue – can uneducated children infer the logic or "program" behind a visuo-motor pattern? Furthermore, the experiments are clever and innovative, especially the second one where participants have to extend patterns in various ways, and some degree of generalization is observed.

We thank the reviewer for this positive evaluation!

Unfortunately, however, as I argue below, both the design and the analyses suffer from problems which imply that the results do not (yet?) support the very strong conclusions that are proposed. Below, I list a number of problems.

We thank the reviewer for these constructive comments, each of which we address at length below.

**Design.** The program inference hypothesis that the authors propose does not seem to be very directly supported by the data. Rather, the authors show that it provides a better fit than the three n-gram alternatives that they propose. I am a bit worried about this approach for several reasons:

1. Do they consider enough alternative models? There is a huge gap between ngram models and the very sophisticated programing language that they proposed. According to Dehaene et al's (2015) hierarchy of sequence knowledge, intermediate levels could involve numerical knowledge and chunking (for instance the children might learn that "blue black" is one chunk and "red black" is another chunk). It seems to me that, at least for experiment 1, such knowledge could suffice to respond accurately and explain the observed behavior. It would be great if the authors could reject those alternative models, but at the very least they should be discussed.

We agree with the reviewer that children could in principle succeed in Experiment 1 using a chunking strategy, in which they learn the meaningful units and replicate them. Identifying the chunks (e.g. AB in ABABAB) would simply require learning the transition probabilities among sequential items, an ability that is available even among infants. Indeed, as we show, a simple bigram model has upwards of 50% accuracy in predicting the next item, well above chance performance (see Figure 3c in revision). Critically, the question we target is not just what models can succeed on the task, but what model best explains the specific patterns they produced, even when they were incorrect. The results are clear in this respect: Children's responses (Figure 2) exhibit structure over and above simple associations of n-grams.

We have elaborated on this important point in the revised discussion, where we say:

*To be clear, we do not wish to claim that children perform program induction in the specific way we have implemented here; Alternative implementations relying on different primitives and different*

*compositional constraints may account for behavior equally well. Nor do we wish to claim that simple association learning (like transition probabilities) is irrelevant to this kind of learning – it is not. Rather, the central claim is that when children encounter new data, they appear to rapidly induce not just the regularities therein, but also the rules, performing an inferential process that is analogous to inducing programs in a language of thought. However it is implemented, this process of program induction can in principle provide a powerful and highly-general learning mechanism that extends well beyond imitation and association.*

That said, we agree that the clearest test of program induction is in Experiment 2, where chunking strategies are not viable for several reasons. For example, whereas a chunk+repeat strategy could produce repeating patterns like those in Experiment 1 (e.g. AABAAB), it could not produce growing patterns (e.g. ABBCCC) or mirrored patterns (e.g. ABCCBA) like those in Experiment 2. Nor could it support generalizations to new or larger alphabets, as we test in Experiment 2. In response to this comment, we now clarify this important point in our discussion of Experiment 1 and the motivation of Experiment 2. Specifically, in the initial submission, we wrote that "These findings show that children's responses reflected sophisticated programs, not randomness or simple associative learning, regardless of culture, education, or numerical knowledge." In the revised manuscript, this sentence has been removed and we now write:

*These findings are consistent with the proposal that children across cultures infer the logical structure of novel patterns using program induction. However, in principle participants could succeed in this task in part by memorizing and reproducing the same set of actions that they observed – imitating their form rather than inferring their abstract structure (perhaps with the help of chunking; 121). To better characterize the learning mechanisms, we conducted a second experiment in which children were given a wider variety of patterns and asked not just to reproduce them, but to generalize them to new lengths and new stimuli.*

We now return to this point in the introduction to Experiment 2:

*Whereas children in Experiment 1 reproduced simple repeating patterns, children in Experiment 2 were asked to generalize complex patterns to new lengths and new stimuli, a better test of their ability to infer abstract logical structure. A new sample of 43 Tsimane' children (ages 7-12 years) viewed each of six novel patterns, presented as static arrays of colored shapes, as shown in the columns of Figure 4a. Whereas the patterns in Experiment 1 were all repeating (e.g. AABAAB), the patterns in Experiment 2 included some repeating patterns, some growing patterns (e.g. ABCCC), and some mirror patterns (e.g. ABCCBA), as in prior work [70, 79]. These stimuli provided a broader test of pattern understanding and, importantly, cannot all be composed by simply repeating a series of actions, learning the transition probabilities among items, or identifying repeating chunks (e.g. AAB). Rather, growing and mirror patterns follow abstract rules but do not repeat.*

2. Even within the low-level models of transition probabilities, the n-gram models tested by the authors may not be the most appropriate. A new idea is the free-energy model or “thick past”, where the idea is that the (temporally discounted) average of recent items is used as a predictor of the next one. This is similar to a 1-gram model, but no longer solely based on the most recent item. Again, I think that this view (or related skip-gram models) could easily account for the data.

> We agree that such models could in part account for participants’ performance in Experiment 1, in which the patterns were repeating (e.g. AABAAB). But they cannot account for the results of Experiment 2, which included growing and mirror patterns. For such patterns (like ABBCCC and ABCCBA), no summary statistic (or even perfect memory) of the initial items can correctly predict the following items. Rather, generalizing such patterns requires inferring a rule like “Each subsequent type has one more item” (for our growing patterns) or “For each item at position i, there is a matching item at position length-i” (for our symmetrical patterns). In response to this comment, we have added this important point to the revised paper (see above).

3. N-grams for n>1 are notoriously difficult to estimate, and here it seems that this is the worst possible situation because the data is extraordinarily limited. Given the difficulty of estimating a 2-gram or 3-gram model from such scarce data, it should not be surprising that the model fit is so poor. At the very least, details of how those n-gram models are implemented would be appreciated.

> We agree, and this is a key point: the data were limited *by design*, as our study sought to address not just how children learn but how they learn *from very limited data*, as they often do in naturalistic settings. In principle, participants could have learned little or nothing from the data we gave them, producing response patterns with no discernible resemblance to the samples. Even given that participants’ responses were non-random, whatever structure they did produce could have been described as well by n-gram models as by program models. Importantly, our design and method do not favor one of these outcomes over the others, and all models were trained on exactly the same data. If n-gram models (or any other) require more data than participants learned from, then such models cannot account for that learning, whereas program models can.
>
> To summarize these points, we have clarified in the paper three aspects of the design: (i) n-grams can explain some of the data but not the bulk of it, (ii) rather, participants inferred abstract rules from small amounts of data, and (iii) this program induction ability might be implemented in many ways.

**Statistics.** On line 272: what do you mean by “we permuted participants’ responses”. Did you permute the labels, i.e. the pairings of response patterns to the 3 stimulus patterns? Or did you shuffle every single response within a pattern? The second choice is inappropriate, as it may overestimate the chance level (if, for instance, participants spontaneously tend to alternate their responses regardless of the proposed pattern; what must be shown is that they produce distinct patterns in different conditions). The massive significance of the results, in

the face of what are, in fact, extremely irregular responses (figure 1), suggests to me that you may have adopted the second approach, which is inappropriate in my opinion. It is less clear to me whether this problem also affects experiment 2, but it might.

In Experiment 1, we permuted the identity (e.g. blue, red) and ordinal position (first, second, etc.) of the items composing each response pattern in order to estimate accuracy scores under random responding, which we have clarified in the revision. Although many chance models are possible, this approach provides a conservative estimate of chance responding because, unlike simply sampling from the set of response options (e.g. unlimited blue and red balls), it samples from the *given* response pattern, effectively shuffling it. Our approach therefore preserves the marginal distributions of items: For example, if in response to AABAAB, a B-loving participant were to produce BBBBBB, this response would *not* differ from chance performance, despite its low *a priori* probability, because any permutation of it would yield the same score. (We note that our n-gram models offer additional – and more sophisticated – models of chance.) Our permutation tests in Experiment 2 worked the same way, with the positions of items shuffled within each response pattern.

To be clear, this permutation technique was used to test whether accuracy in each task differed from chance. The reviewer suggests we test an additional question – whether participants "produce distinct patterns in different conditions." This is a good suggestion and we have implemented these new analyses for both experiments in the revised manuscript. In the results of Experiment 1, we now report:

*In principle, participants could perform above chance even while producing the same pattern in response to different sample patterns. We therefore tested the specificity of participants' responses to each sample pattern, using edit distance (i.e. Damerau–Levenshtein distance) as a measure of pattern similarity. This technique computes the minimal number of deletions, additions, or substitutions required to transform a response pattern into the sample pattern. Results showed that in both groups and numerical-knower levels, participants' response patterns were more similar by this metric to the given sample pattern than to the other sample patterns ($ps < .0001$; see Supplementary Information for detailed results). In sum, responses were both accurate and specific to the given pattern.*

Likewise in Experiment 2, we report:
*To test the specificity of participants' responses to each sample pattern, we also computed the edit distance of each response pattern to the nearest target response for each of the* other *comparable sample patterns (i.e. the ones participants were not looking at). Overall, responses were significantly more similar to the given pattern than to the other patterns ($p < .0001$; see Supplementary Information).*

In the Supplementary Information, we include the details of these analyses and results, including two new figures which visualize the edit distance within and between comparable sample patterns.

**Other minor comments:**
- Line 232: in experiment 1, were the three patterns presented in fixed or in random order? If the latter, can you exclude that the later responses include a significant component of "meta-learning" about the types of patterns allowable in the task? See eg. Participants 207 or 244 who are, surprisingly, performing better on the most complex pattern (Hitch).

> The patterns were presented in a fixed order but we see no evidence of meta-learning. In the last pattern of Experiment 1 (Hitch), accuracy is lower on average than in the preceding patterns. Likewise, we see no evidence of improvement in accuracy (i.e. edit distance) over the 6 patterns in Experiment 2 (which were also presented in a fixed order). One reason we might not expect much meta-learning is that participants received no informative feedback on their responses; All complete responses were treated the same.

- Line 236. It took me several readings to understand what was meant by the Hitch description (I initially thought that the black ball, conventionally, depicted either the blue or the red). Perhaps this sentence could be improved: "we sorted one pair of colors while including a third color at each step"

> We have modified this sentence to read "we sorted two colors while including a third color at each step." Hopefully, the figure and in-line colors help to clarify this as well.

**Reviewer 2** ###############

Overview: This is a wonderful paper, and will be an important addition to a wide range of literature in cognitive science. The topic is of broad enough interest to merit publication in Nature Communications, the conclusions is more than interesting enough, and the methods are robust.

I often give pages and pages of referee comments, but have very few comments here as I found the paper to be edifying, clear, and excellent all around. Here are the questions I would most like the authors to answer.

> We thank the reviewer for these positive comments!

1) Although it seems like a side comment, the claim about Tsimane not having standardized words for geometric shapes seems hard to believe. (The authors write "Many Tsimane' have little or no formal schooling, do not have standardized words for basic geometric shapes (like circle, square, and triangle), and many adults do not read, write, or use math." There is a Butterworth et al. citation on the sentence before this claim—is that what is supposed to substantiate the standardized word claim? I know this seems like a small point but part of why I think the paper should be accepted is that it is just very rigorous and believable (which is hard in field work and

often not quite true of papers in Science, PNAS). So I would like a little more substantiation of this claim, as it was the only one in the paper that I found to be quite unintuitive.

> This claim about geometric shape naming is based on pilot data one of the authors collected among Tsimane' adults. Since those findings are provisional (and we are aware of no other such findings in the literature), we have removed this phrase from the quoted sentence. Thank you for bringing this detail to our attention. Perhaps future work will better document differences in geometric shape categories.

2) Is there a difference between program induction and learning a rule in the language of thought? The more I think about it, the less daylight there seems to be here. Some discussion on what the differences are, or how to pull it apart, could be edifying.

> We agree that program induction (as we characterize it) is a kind of rule learning in a language of thought - specifically learning by Bayesian inference. We hope we have clarified this relationship in an addition to the Discussion, which says "the central claim is that when children encounter new data, they appear to rapidly induce not just the regularities therein, but also the rules, performing an inferential process that is analogous to inducing programs in a language of thought."

3) Why think the second part of this sentence? "This pattern of results suggest that children across cultures and ages can infer the structure of novel patterns even before they have learned to count, and that this understanding does not reflect a single moment of insight, but rather a piecemeal process of inferring the individual algorithmic features of the input." It goes against so much of learning, which is often not stepwise (not just one-shot word learning either; see, e.g., the Gallistel and King 2009 book).

> We do not mean to say that learning is perfectly continuous: There are certainly marked discontinuities in children's abilities. But some theories posit sudden turning points or an individual "crucial induction" (Carey, 2008) in cognition. Even when this assumption is not stated explicitly, it is implicit in the many studies – including many studies of pattern learning and numeracy – that judge understanding in a binary way, for example asking whether children do or do not have theory of mind, understand the logic of counting, or know the meaning of a given word. In principle, participants could have produced one of two types of responses in our tasks, either a correct answer (that respected the logical relations in the sample pattern) or a random answer (i.e. pure noise). Instead, we found that children successfully reproduced some aspects of the patterns, while ignoring others, suggesting that this type of learning is not all-or-nothing, at least in tasks like ours (in which they are faced with small amounts of unexplained data). In response to this comment, we have added some verbiage to this paragraph of the paper in hopes of clarifying our meaning.

4) I'm also wondering how much of the anti-nativist implicature here is on purpose. Why are these results inconsistent with nativism (which isn't said, but is implied)? Is it that they are the output of a domain-general learning mechanism? I would've thought the evidence here was equally suggestive of a set of prelinguistic, universally shared conceptual primitives. At the very least the paper is fairly anti-Whorfian, and it could be worth noting that (at least for theorists like Gary Lupyan who think that Whorf is back, and true). Maybe I am misreading and the implicature isn't really there? Perhaps it would be best to include a short section or paragraph on how you think these results interact with debates about nativism.

We thank the reviewer for these interesting questions.

First, on nativism. As we see it, the question of nativism vs. empiricism is how much of cognition is innately specified and how much depends on experience. Many accounts of human development are strongly nativist in that they posit innate, domain-specific cognitive content and/or learning mechanisms (e.g. in math, physics, language). A less nativist account posits that what is innate is a more general learning mechanism, one that isn't tailored to specific domains. We believe our results provide evidence that one such domain-general mechanism is available across cultures without extensive schooling, which in principle could obviate the need for domain-specific learning mechanisms. In this way, our claims are indeed anti-nativist, as we clarify in the revised discussion:

*To be clear, these findings do not rule out domain-specific learning mechanisms, which could complement domain-general mechanisms in domains like natural language, social relations, and spatial reasoning. However, such mechanisms may not be necessary to explain how humans learn specialized cognitive abilities from limited input. For example, one need not posit an innate neural mechanism for learning the counting algorithm [92] given that children can learn a wide variety of such algorithms in minutes, even before they have learned to count. Rather, many of the specialized skills people acquire in a given setting — from counting to cultivating crops to parallel parking — may be algorithmic abilities learned via domain-general learning mechanisms.*

Second, on Whorf. We are unsure why the paper strikes the reviewer as anti-Whorfian, as this is not a position we wish to imply. On the contrary, to the extent that human cognition is flexibly shaped by the structure of the environment (e.g. via program induction), differences in the linguistic environment should produce differences in cognition, a Whorfian claim to which we are sympathetic.

Also in the Introduction, you are missing a 'to' in the second sentence (062)

Thanks, fixed.

But these claims shouldn't detract from the main point: its an excellent paper, full of difficult to gather data.

Many thanks!

**Reviewer 3** ##################

The authors present an intriguing argument for and novel (as far as I'm aware) data bearing on the possibility of domain-general program induction as a human-universal cognitive mechanism which underpins human-unique learning abilities.

The methodology and analysis of this work seems solid and I particularly commend the effort to work with atypical, hard-to-reach populations.

Thank you!

However, it seems to me that several gaps remain within the current manuscript - both in its overall logic and in the connection between the evidence presented and the conclusions the authors wish to draw:

1 - The notion that program induction may support human-unique learning is emphasized strongly in both introduction and conclusion of the piece. At the same time I see no reference to evidence that non-human animals are unable to complete the kind of inductive tasks presented here. I suggest that the authors either provide such evidence, or drop the claim that the mechanisms they are studying are necessarily human-unique. The point is hardly trivial - after there is an extensive literature showing that non-human animals struggle with, but are ultimately capable of, abstract relational reasoning (e.g. relational matching).

We agree with the reviewer that program induction is not necessarily unique to humans. Indeed, we suspect it may be found in a variety of animal species. To clarify this important point, we have written a new paragraph in the Discussion:

*Although program induction may support many human-unique cognitive abilities, it may not be unique to humans. Many non-human animal species show evidence of flexible learning, abstraction, and generalization [125–128], even in domains unlikely to be the target of natural selection [129], and at least some of this learning may reflect program induction. For example, although slower than humans, rhesus macaques learned the latent recursive structure in novel patterns and generalized it to new stimuli [130]. Whereas some accounts attribute humans' extraordinary cognitive abilities to qualitative differences in learning mechanisms [53, 62], others attribute them to quantitative differences in information processing capacity [131]. In model systems, differences in memory alone can have profound impacts on what can be learned, holding constant the amount of data and the inferential mechanisms. In this way, program induction could be fundamental to learning in humans and non-human animals alike, despite dramatic differences in their cognitive abilities.*

2 - Is it not possible that the kind of patterns presented here could be processed by an initially domain specific mechanism? I have in mind the ability of children to flexibly learn language across any modality (sight, sound,

touch). Given that language-learning requires much more complex program induction than the present work, in what way can we distinguish the present results from yet another expression of the remarkable human ability to learn systems of symbolic communication?

> We thank the reviewer for raising this important question about domain specificity. The answer depends on how one defines "domain." If the language domain is construed broadly enough that it encompasses any rule-based pattern in any modality, then our results could be explained by a learning mechanism specific to that domain. But such a domain would be extremely broad, including not just speaking and signing, but many other skills as well, including square dancing, counting, and tying shoelaces. Such a domain might better be called "pattern learning" than "language learning." Whether we classify these various skills as one broad domain or many narrower domains, the implication is the same: Learning them may be powered by the same underlying inferential process, one that is available to children across widely different cultures.

The task is, after all, quite clearly a communicative context. For sake of illustration, if we presented schooled, Western individuals with a task in a repetitive, unfamiliar language and asked them to extend the patterns therein - would we not also find the kind of noisy-but-program-like responses reported here?

> We agree that people would likely be able to extend novel patterns in an unfamiliar language, just as they did in our shape-patterning task. Indeed, there is some evidence of this ability in prelinguistic infants (e.g., see Marcus et al., 1999). The question is how do people make these sorts of inferences, and how generally? Our studies addressed this question in a task that was not linguistic or communicative. (Although the context of any experiment is in some way communicative, our tasks were not: Sample and response patterns were meaningless and participants did not receive rewards or feedback as a function of their responses.) And the results suggest that the learning mechanisms that power learning in any individual domain may be general and flexible enough to power learning in any other. We also note that recent work shows that the brain's language systems do not appear to be involved in many forms of thought, including symbolic thought like math (see Fedorenko, Piantadosi, & Gibson, 2024).
>
> That said, we agree that our results do not rule out a role for domain-specific learning mechanisms, a point we have clarified in the revised manuscript:
>
> *To be clear, these findings do not rule out domain-specific learning mechanisms, which could complement domain-general mechanisms in domains like spatial reasoning, social relations, and natural language [140]. However, such mechanisms may not be necessary to explain how humans learn specialized cognitive abilities from limited input. For example, one need not posit an innate neural mechanism for learning the counting algorithm [101] given that children can learn a wide variety of such algorithms in minutes, even before they have learned to count.*

3 - The analyses, as they are presented, do not adequately control for the effects of formal schooling. Specifically, while the authors report data from children with no school experience in Experiment 1, no such data are reported for Experiment 2. This is a crucial point given that the pattern-following in Experiment 2 is, by the authors own admission, crucial for attributing program-induction. My concern is that decades of evidence in cross-cultural psychology has shown that exposure to even minimal amounts (e.g. one year) of schooling can dramatically change patterns of response on cognitive tasks, including those involving abstract patterns (see e.g. Luria, 1976; Sharp et al., 1979). Without data from unschooled participants in Experiment 2, then, it is difficult to be confident in the authors assertion that "Presented with small amounts of data, children across cultures can induce the abstract logical structure therein, constructing ad-hoc explanatory theories without... formal schooling."

> We thank the reviewer for this important point. We agree that the stronger evidence for program induction comes from Experiment 2, in which all participants reported at least some schooling. We have therefore moderated our claims about the presence of program induction in unschooled participants throughout the paper. Rather, we focus on children's ability to induce algorithmic structure "without instruction, knowledge of common algorithms, or large amounts of data." We note that although participants in Experiment 2 had some schooling, for many of them this schooling was not sufficient to produce basic counting knowledge, an algorithmic domain specifically targeted by schooling. Yet even these subset-knowers succeeded in our tasks.
>
> In response to this comment, we have also added a new paragraph to the Discussion about the role of cultural practices as well as formal and informal instruction in program-induction abilities:
>
> *Many of our participants were young, lacked extensive schooling, and understood little about common algorithms like counting, but they all were practiced in pattern recognition, including in language, physical reasoning, social inference, and a variety of cultural practices involving patterns. For example, Tsimane' children may participate in fishing, harvesting, and preparing food [27]. Such activities provide children with ample opportunity to infer the implicit rules that govern their environment, starting from birth, and may therefore improve program-induction abilities, even if they are innately available. These abilities may also be sharpened by formal and informal instruction [122–124], in which teachers and caregivers make explicit the algorithms underlying cultural skills like adding fractions, tying shoelaces, or building canoes. In this way, even passive participation in cultural groups may hone children's ability to induce algorithmic structure from data...*

4 - I have a hard time interpreting Fig 4: In commonsense terms, what does it mean for participants to be 'above chance' in replicating the patterns?

In short, being “above chance” here means being closer to the right answer than we would expect if participants were responding randomly.

Specifically, we computed accuracy using edit distance, which just counts the number of additions, deletion, or substitutions that would be required to transform a given response pattern (e.g. ABABBB) into the nearest ‘correct’ response (e.g. ABABAB). This measure is nice in part because it provides a continuous measure of similarity between two patterns, rather than a simple “correct” or “incorrect” outcome. To operationalize chance performance*, we just randomly scrambled the positions of the items in each response pattern (e.g. ABABBB -> BABBAB) and then computed the edit distance again. By doing this kind of permutation for every response ten thousand times, we get a distribution of the edit distances we should expect if participants were making random arrangements of items. We use this distribution to define the y-axis of Figure 4a (5a in the revision), where each unit is equal to the standard deviation of the chance distribution. By definition, 95% of the chance distribution lies within the skinny grey bar (which ranges from -1.96 to +1.96). Points below this region therefore show response patterns that are closer (i.e. less distance) to a correct answer than would be expected by chance. One of the points falls above the grey region, showing that for that case, response patterns were farther from (i.e. more distance) a correct answer than would be expected by chance. This anomaly likely reflects the fact that participants often produced alternating patterns in response to this mirrored pattern.

*Although many chance models are possible, this approach provides a relatively conservative estimate of chance responding because, unlike simply sampling from the set of response options (e.g. unlimited blue and red blocks), it samples from the *given* response pattern, effectively shuffling it. Our approach therefore preserves the marginal distributions of items: For example, if in response to AABAAB, a B-loving participant were to produce BBBBBB, this response would *not* differ from chance performance, despite its low *a priori* probability, because any permutation of it would yield the same score.

Also, my suspicion is that chance is a very poor baseline here. After all, I'd bet that most humans, when given a series of geometric shapes to arrange, will make some kind of pattern out of them (given that all the cultural groups I can think of have some kind of geometric decorative motifs, more on which below). If this is the case, it is possible that 'above chance' performance is driven in whole or part by individuals just arranging the shapes into patterns of their own choosing. This would also drive the 'program-like' result displayed in 4b. Of course I may be missing a reason why such an interpretation is untenable - but if so I would appreciate a brief discussion by the authors.

We agree that humans naturally pattern-makers and pattern-*finders*: They discover the patterns in the things they observe. The question is how and how general these abilities are. To address these questions, we measured two things about the patterns children made: Algorithmic regularity and accuracy. Our measure of algorithmic regularity indexes how structured responses were - and how well this structure can be characterized by simple algorithms. As the reviewer correctly points out, participants could score high on this measure even if they strayed wildly from the sample patterns. We therefore also measured

their accuracy, which indexes how well participants' response patterns reflected the *specific* sample patterns they were given. This measure is especially intuitive in Experiment 2, where accuracy was measured as the edit distance (i.e. number of additions, deletions, and substitutions) to the nearest "correct" answer, shown in Figure 4a (5a in the revision). Importantly, participants cannot perform above chance on this measure by simply making any pattern they wish. Rather, they must make a pattern that reflects the structure of the sample pattern. In this way, this measure shows that "Response patterns shared algorithmic structure with the sample patterns they observed." We have clarified this important point in the revised manuscript.

In response to this comment, we now also include an additional measure – which we call "specificity" – which indexes how specific participants' responses were to a given sample pattern. They scored high on this measure in both experiments, providing additional evidence that participants were making responses specific to each sample pattern. We clarify this important point in the revised results of Experiment 1:

*In principle, participants could perform above chance even while producing the same pattern in response to different sample patterns. We therefore tested the specificity of participants' responses to each sample pattern, using edit distance (i.e. Damerau–Levenshtein distance) as a measure of pattern similarity. This technique computes the minimal number of deletions, additions, or substitutions required to transform a response pattern into the sample pattern. Results showed that in both groups and numerical-knower levels, participants' response patterns were more similar by this metric to the given sample pattern than to the other sample patterns ($ps < .0001$; see Supplementary Information for detailed results). In sum, responses were both accurate and specific to the given pattern.*

...and in Experiment 2:

*To test the specificity of participants' responses to each sample pattern, we also computed the edit distance of each response pattern to the nearest canonical response for each of the other comparable sample patterns. Overall, responses to a given pattern were significantly more similar to that pattern than to the other patterns ($p < .0001$; see Supplementary Materials).*

In the updated Supplemental Information, we include the details of these analyses as well as two new figures. In short, these new analyses show that participants were not just making "some kind of pattern" but rather were inducing the structure of the specific pattern they were given.

5 - Relatedly, the authors emphasize that the task children are presented with is completely unfamiliar to them. But in what sense are they new? And how do we know this? It is obviously the case that the specific set of stimuli and instructions will be new - as they would be to any participant who had not completed this specific task. Consequently the 'novelty' must be at the level of 'this type of task'. But why should we assume that Tsimane

children are unfamiliar with this kind of task? For instance, (unless I'm misremembering) the Tsimane use geometric patterns for face painting - in many cases using the very same shapes and colors (e.g. red squares, black circles) as the tasks presented here. Let us assume (I think plausibly) that the patterns each individual paints are neither exact repetitions of previous instances, nor totally random. From this it follows that what individuals have learned is the ability to replicate some abstract scheme or aesthetic. Is this not familiarity with something like the present task? If not, why not? More generally, what is the type of task used here such that we can be confident that Tsimane participants will not be familiar with it?

We agree – Tsimane' culture includes a variety of customs involving visual patterns (e.g. weaving, face-painting), perhaps even of colored shapes, some of which may be well known to our participants. These culturally-embedded patterns may have prepared participants for our experiment in some way, an important point we now discuss in the revised paper (see below). However, they do not pose a problem to our arguments so long as they are distinct from the specific patterns we tested in *either* their visual form or logical structure. First, if participants had experienced our specific stimuli (e.g. red squares, black circles) used in other patterns, this experience would only interfere with their ability to correctly induce the correct pattern, a potential source of noise. Indeed, a large literature shows that superficial visual features of stimuli often interfere with children's relational reasoning in this way (e.g. in relation-match-to-sample tasks; Gentner, 2005). Therefore, participants' success in inferring the correct algorithm cannot be attributed to familiarity with the stimuli.

Second, if a pattern's logical structure (e.g. indefinite alternation as in ABAB...) was familiar to participants (e.g. from sorting two kinds of seeds), the inferential challenge posed by the sample pattern – inducing the rule that produced the sequence – would be largely the same challenge, as long as the pattern's visual form was novel *because all they get is the form*. In order to make analogies to other sequences/arrays with similar abstract structure (like sorting seeds), participants must first infer that abstract structure. In short, if observable features were familiar, this would only degrade abstraction, and abstract features could be familiar only if they can be induced from the observable form. Therefore, only if participants were familiar with *both* the observable form and abstract structure of a given pattern – that is, the same tasks – would this present a problem for our conclusions. In response to this comment, we now clarify this important point in a new paragraph in the revised Discussion:

*In principle, cultural experience could allow children to reproduce familiar patterns without inferring their abstract structure, but this possibility cannot easily explain participants' performance in our experiments, for three reasons. First, if the colors and shapes of our stimuli (e.g. red circles) were familiar to participants from other activities – and they likely were – participants might try to generalize from those activities to our tasks. However, assuming that these shapes/colors played different roles in those activities, this prior experience would be a source of noise in our experiment, potentially interfering with participants' ability to correctly reproduce or generalize the given pattern, rather than facilitating it [125]. Second, some cultural activities could share abstract algorithmic structure with our sample patterns, despite superficial*

*differences in appearance. For example, patterns like Sort and Split may be familiar to children who have distributed objects among two parties and patterns like Mirror.4 may be abstractly analogous to balancing objects on a scale. Yet, such similarities in abstract structure cannot explain success in our task because participants observed the form only – the abstract structure must be inferred in order for any structural analogies to be made. Finally, whereas it is unlikely that our participants had already encountered the specific sample patterns we used, it is even less likely that they had already generalized those patterns as they did in Experiment 2, translating them to new stimulus sets, larger alphabets, and greater lengths. In this way, children's everyday cultural experience likely facilitated their performance in our tasks, but only insofar as it improved their pattern induction abilities in general.*

In sum, the methodology and findings of the present work are intriguing. The ambitious scope of the argument is also a good match for Nature Communications (e.g. "program induction may be central to both our cognitive flexibility and cultural diversity, allowing people to discover and generalize the latent algorithmic structure of their natural and cultural environment, whatever it may be").

Thank you!

That being said, I am not yet convinced by either the logic of the authors' claims, or by the way the data they present supports these claims. I suggest that a substantial revision is in order for the manuscript to be accepted.

We thank the reviewer for the helpful questions, comments, and insights. They have led us to substantially clarify the logic of the experiments, to add a new test of response specificity to the results, and to better contextualize the findings in an expanded Discussion.

**Minor notes:**
- It would be useful for the authors to discuss how exactly they translated their instructions to recreate 'the same pattern'. In other small-scale languages I am aware of there is no easy translation of this term.

Yes, we avoided the word "same" and instead asked the children – who generally have many siblings – to make a "sibling" pattern. As we say in the paper, "Starting from scratch each time, participants were asked to look closely at the sample pattern and make its 'sibling,' a new pattern that was as similar as possible to the sample, given the items available and length required." We have now added: "This wording avoided the use of words like ``same," whose meaning varies across cultures and contexts."

- Figure 3a is crucial to illustrating the program-induction argued to drive the present results. Yet, the key element of this figure is a "modal response" from Tsimane participants. This feels needlessly obscure to me - leaving open the possibility that e.g. the modal pattern was repeated twice - while all other responses were unrelated to the presented pattern and just abided by some pattern of their own (which would drive the Fig 4b data).

Indeed, these modal responses accounted "for as much as 98% and as little at 12% of responses to a given pattern." We agree that this is perhaps too large a range to be helpful to this figure, and so, in response to this comment, we have modified Figure 3a (4a in the revision) so that the responses in the grey strips now show an example target response for each sample pattern and condition rather than the modal response (although these were often the same). We hope that this change will clarify to readers the kinds of generalizations that Experiment 2 required of participants.

To be clear, this figure is not meant to clarify how participants' responses related to the sample pattern. That important question is answered in two ways: (1) by our analysis of response accuracy (see Figure 5a in the revision), and (2) by our new analysis of response specificity.

It would be extremely helpful to have, in supplemental information, the full set of responses for both Experiment 1 and 2 displayed in the style of Fig 1.

We have added two new figures to the Supplementary Information, which visualize the full set of participant responses for each experiment.

**Reviewer 4** ################

The manuscript entitled "Algorithm induction in indigenous Amazonian children" explores the cognitive mechanisms of learning using a program induction framework. Thanks to two experiments conducted among children coming from an Indigenous community (the Tsimane' in Bolivia) and from the USA, the authors bring evidence that children can infer patterns early in life and may learn novel patterns more effectively through induction than imitation or substitution. Supported by rigorous methodologies and a rich dataset, this work has the potential to make a meaningful contribution to the field of cognitive development, particularly in understanding how children learn across diverse cultural contexts.By the innovative aspect of their work and the implication for our understanding of human cognitive development, there is no doubt that this work is a relevant contribution to the field and meaningful for Nature Communication journal.

While the study is insightful, several critical concerns must be addressed before it can be considered for publication. Three main aspects should be specifically addressed: 1) the lack of a rigorous ethical demarch; 2) the justification of working with north American children; and 3) the extent of the interpretation. Additionally, the manuscript's accessibility to non-specialists could be improved by reducing jargon and expanding explanations. Below, I have detailed my main concerns, as well as minor comments and suggestions, that, I hope, will help the authors to review this work.

**Main concerns.**
1) Lack of a rigorous ethical demarch.
My main concern relates to the ethical approach taken by the authors and encompasses several aspects of the study.

**Ethical Approval Documentation**
First, the ethical approval obtained from institutional and local organizations should be explicitly stated, including their approval numbers. Was ethical approval obtained from the Bolivian National Ministry of Science? If not, this omission should be clearly justified.

> Although the Academia Nacional de Ciencias de Bolivia has worked to promote science in Bolivia since 1960, to our knowledge it plays little role in setting or approving ethical standards for research. Indeed, our Tsimane' collaborators and we are aware of no such body at the national level. For our studies, we therefore sought and received approval from the Gran Consejo Tsimane', a council of Tsimane' elders who oversee research projects in Tsimane' villages from ours and other groups. We also received IRB approval from our home institution, UC Berkeley, and now report its protocol number.

**Informed Consent Procedures**
In addition to institutional ethical approval, the authors should have obtained Free, Prior, and Informed Consent (FPIC) from every individual involved in the study. Currently, the manuscript does not clearly specify whether or how informants' consent was obtained. The authors state that they "received assent from all participants and consent from their legal guardians," but further details are necessary. How was the research presented to the children? What measures were taken to ensure that participants fully understood the study's aims, methods, risks, benefits, and data usage? How was children's consent obtained?

Clearly articulating the ethical procedures is essential for any research involving human participants, particularly when working with Indigenous Peoples and children. Research with children requires adherence to specific guidelines on presenting the research and obtaining consent. For example, see Graham, A., Powell, M. A., & Taylor, N. (2015). Ethical research involving children: Encouraging reflexive engagement in research with children and young people. Children & Society, 29(5), 331–343.

> We thank the reviewer for raising these important concerns and agree that the initial manuscript did not sufficiently specify the ethical protocols we followed for these vulnerable populations. In response to this comment, we now detail our protocols for recruiting and compensating participants and for obtaining their free, prior, and informed consent. As we write in the revised manuscript:
>
> *Tsimane' children (N = 73; ages 3 - 13 years; mean age = 7.5 years, mean schooling = 1.4 years) were tested in their villages along the Maniqui river of lowland Bolivia. Villages were selected according to their accessibility from San Borja, in consultation with el Centro Boliviano de Investigación y Desarrollo Socio*

*Integral (CBIDSI), a Bolivia-based NGO specializing in the study of Tsimane' health and behavior. Tsimane' participants consisted of volunteers presenting themselves at the village schoolhouse. As many Tsimane' adults do not read or write, all consent procedures and instructions were conducted orally by a translator who grew up in a local community, as approved by the UC Berkeley IRB. The Tsimane' translator explained aloud in Tsimane' the purpose, risks, benefits, duration, and voluntary nature of our study, as well as the age range of interest. Potential participants were encouraged to ask questions. Parental consent to child participation and data usage was documented by the research team. Given parental consent, we showed children the experimental stimuli and asked them if they would like to play a game with us using those stimuli. A positive response constituted assent and researchers intermittently re-requested assent during testing. Parents were invited to sit nearby participants for their comfort, but to abstain from assisting in any way with the tasks. Parents and children were compensated with small gifts, regardless of their performance or completion...*

*US children (N = 24, ages 3:1 - 5:5; mean age = 4.4 years, mean schooling = 1.4 years) were tested in preschools affiliated with UC Berkeley in Berkeley, California. Parents were provided with a written description of the study's purpose, risks, benefits, duration, and voluntary nature. A subset of these parents provided advance written consent for their child's participation and data use. Of these children, participants were chosen in consultation with the preschool teachers on the basis of age and history of research participation (to limit their frequency of participation). US child assent was obtained as among Tsimane' children. Participants were gifted a sticker regardless of performance or completion.*

*All experimental protocols were approved by the Institutional Review Board at the University of California, Berkeley (Protocol #2018-06-11209) and by el Gran Consejo Tsimane' (The Tsimane' Grand Council), which oversees research in Tsimane' communities.*

**Researcher Positionality**

The positionality of the authors who conducted the data collection should be discussed. Potential biases and limitations arising from differences in age, culture, or gender between the researchers and the children involved in the study should be explicitly addressed.

The reviewer correctly notes that the demographics of some of the researchers were different in many ways from those of the participants, notably in age, education, culture, language, power, and wealth. Such differences can produce biases in the responses of participants and/or in the interpretation of results by researchers. Although no study may be immune to such effects, ours guarded against them in several ways. In response to this comment, we now outline these risks and our efforts to mitigate them in a new subsection of the methods, called "Mitigating cultural bias," where we write:

*We took several measures to limit any effects of researcher bias on our cross-cultural findings (in both experiments). First, our Tsimane' research was conducted in partnership with el Centro Boliviano de*

*Investigación y Desarrollo Socio Integral (CBIDSI), a Bolivia-based NGO specializing in the study of Tsimane' health and culture, whose expertise informed selection of villages, participant recruitment, informed consent, compensation, and task design. During Tsimane' data collection, US American experimenters were accompanied by native Tsimane' research assistants who acted as the primary touch points with participants and provided them with instruction in their native language. Second, to mitigate misunderstanding of the tasks, we provided demonstrations, practice trials, and comprehension checks to evaluate participants' understanding behaviorally before performing critical trials. Third, our outcome measures were standardized across individuals and groups, did not include informative feedback, and focused on observable features of participants' behavioral responses, leaving little room for interpretation.*

**Contextual Embedding**

The description of the Tsimane' community is overly brief and outdated. The study should be better contextualized within the realities of the communities involved. Currently, the manuscript relies on a single, outdated reference to describe the population, reducing the description of the Tsimane' to approximately six lines. This brevity not only creates a gap in the manuscript but also poses challenges for interpreting the findings within the appropriate cultural context. A more comprehensive account, supported by recent literature, would provide a richer and more accurate foundation for interpreting the results.

We thank the reviewer for bringing this point to our attention. We agree that our description of the cultural context was insufficient in the original submission. In response, we have updated and expanded our description of the Tsimane' context, including many new citations and a new figure – Figure 1* – depicting this unusual testing context photographically. As we write in the revised paper:

*We focused our experimental work on a population which provides an especially strong test of universality: indigenous Tsimane' children in lowland Bolivia. The Tsimane' are a small-scale society of the Amazon basin, where they rely primarily on hunting, gathering, fishing, and farming for subsistence (see Figure 1; 89, 90). Although formal schooling is now available in many villages, the regularity and quality of this schooling is highly variable, as in other indigenous groups. As a consequence, many Tsimane' adults have little or no formal schooling and do not read, write, or use math [91, 92]. Tsimane' children spend much of their time outside, exploring large swaths of the forest on foot with other children [93]. They acquire a wide variety of skills before reproductive age, including in food production (e.g. fishing, hunting, gardening), building and crafts (e.g. making houses, textiles, and canoes), and childcare (the average Tsimane' has 9 siblings; 27, 94, 95), as well as rich ethnobotanical knowledge [96, 97].*

*Note that the addition of this figure in the revision changes the numbering of all the subsequent figures.

2) The inclusion of North American children.
While the authors present and use data from two different settings, they do not clearly articulate the comparative aspect of this research, nor do they fully leverage the comparison to better interpret their findings. For instance, although US children appeared to have lower accuracy scores than Tsimane' children in Experiment 1, the authors did not discuss this difference.
The data from US children is limited to Experiment 1 (see lines 822–830), and the sample size is three times smaller than that of the Tsimane' group. Furthermore, the individual characteristics of US children are not thoroughly considered but rather assumed. Factors such as schooling, familial, social, and cultural contexts, as well as health, may significantly affect cognitive development. For the Tsimane', schooling is assumed to be low, but how was this assessed? What kind of data did the authors use as a proxy for schooling?

> We assessed schooling among Tsimane' children by asking parents to report years of schooling. Our tests of number knowledge also serve as a proxy for formal education in both groups. We clarify these points in the revised Methods.
>
> We agree that there are many important questions about potential cross-cultural differences in these inductive abilities, and we hope that future research will investigate which specific cultural or demographic factors are most important for this type of learning. However, our motivation for including these two populations was not to test effects of specific cultural or demographic features (like subsistence style, social structure, or health), but rather to test the generality of our effects across large cultural differences. We have clarified this important point in the revised manuscript:
>
> *In many ways, the experience of Tsimane' children differs dramatically from that of urban US American children, including the ecological setting, subsistence style, social scale, level of industrialization, language, and material culture. Although no one of these differences was of specific interest, these distinct populations allowed us to test whether participants' inductive abilities were robust to large cultural differences.*

Given the reduced sample size and the data being restricted to Experiment 1, is it sufficient to support generalizations? This study could undoubtedly provide strong evidence of cognitive mechanisms related to human learning processes without including the US sample. Such a focus might also encourage further research in other cultural and social settings. Simplifying the analysis to include only the Tsimane' data would better highlight the study's key insights and improve clarity.

Therefore, I question the relevance of including data from US children. What does it contribute to the overall conclusions of the work? The comparative aspect of this study should be better justified, clearly presented in the results, and fully interpreted in the discussion. Alternatively, the authors might consider focusing solely on the Tsimane' sample, which would allow them to situate their study more effectively within the cultural and social-ecological context of the children they are studying. This would also simplify the manuscript, which is currently dense and complex.

We agree that the second experiment alone could in principle stand on its own. However, we continue to feel that the US sample is valuable for two reasons. First, it provides a conceptual replication of our primary results in two independent samples. Second, it speaks to the generalizability of the results, as results from any single population (even if it is underrepresented in the literature) may be limited in their generalizability. Children's success across two very different cultural contexts suggests that the underlying learning mechanisms may be universal, or at least highly regular.

**3) The extent of the interpretations.**
The discussion section is concise but lacks depth and broader contextualization. The discussion might be reviewed to better structure it around clear insights and to extent the insights provided by this study not only for the field of cognitive development at the theoretical or methodological insights, but also regarding what these insights bring to human development in general, or to Tsimane′ learning process and livelihood. Also, if the comparative aspect of the study is maintained, it should be further discussed.

In revision, we have substantially expanded our discussion of the results and their implications for theories in cognitive development, experimental psychology, comparative psychology, and education. In short, the findings suggest that the richness and diversity of human skills may be driven by a fundamental learning mechanism that is broadly shared across human groups.

Finally, the language used in the manuscript might be improved to avoid employing too often specialized "jargon" and to rather try to open the work to a broader academic audience.

We apologize for the jargon and have tried to simplify and clarify whenever possible in the revised manuscript.

**Minor comments:**

Introduction
Clarify ambiguous phrases:
- "people learn so much from so little" Specify what "so little" refers to.
  To more clearly convey the meaning of this phrase, we have replaced it with "To explain the astonishing speed of human learning..."

- "basic domains of knowledge" provide examples to clarify it
  This phrase refers to the preceding list of examples, including "language, object perception, causal inference, spatial relations, geometry, numerical perception,"

- Why assuming observation is "simple"?

We have removed the word "simple."

- Please explain what "improvisation intelligence" refers to

This term has been used in contrast to "dedicated intelligence" and has been described as "a suite of uniquely flexible cognitive capacities that allow our species to acquire locally adaptive behavior in a wide range of environments" (Boyd, Richerson, Henrich, 2011). We use this term to summarize the contents of the preceding paragraph, including the same sentence, where we write "This extraordinary cognitive flexibility has allowed our species to populate a wide variety of natural environments around the world and to innovate new technological, social, and political tools but....this 'improvisational intelligence'..." We refer readers to the papers cited for a full discussion of these ideas.

Paragraph from Line 115 to 141: Adding age range to the statements formulated would help to situate the insights brought. For instance, specific what is understood here by prelinguistic infants (what is the age range)? What children and infants mean here (both categories might largely differ depending on the cultural and social contexts)?

We now specify the age of participants in the most relevant study cited, replacing "prelinguistic infants" with "seven-month old infants."

Most importantly: the description of the Tsimane´ should be much more developed and nourished by updated references. A large amount of research has been conducted among the Tsimane´, including studies on memory, learning process, and schooling. Please update this information and expand this paragraph to better situate the study within local realities.
Consult the work made by Gurven and colleagues, and for instance the following ones:

- Schniter et al., 2015 Skill ontogeny among Tsimane forager-horticulturalists https://onlinelibrary.wiley.com/doi/abs/10.1002/ajpa.22757
- Reyes-García et al 2016 Schooling, Local Knowledge and Working Memory: A Study among Three Contemporary Hunter-Gatherer Societies https://journals.plos.org/plosone/article?id=10.1371/journal.pone.0145265#pone.0145265.ref045
- Gurven et al., 2017 Cognitive performance across the life course of Bolivian forager-farmers with limited schooling. https://psycnet.apa.org/record/2016-41992-001

We thank the reviewer for pointing out the paucity of relevant citations on Tsimane' culture and demography in our original submission. We have expanded our discussion and citations thereof in the revised submission, including the three papers suggested here.

Results

Some aspects of the results might be moved to the Discussion part in order to gain in clarity. Consider moving interpretive statements, as for instance the sentences in Line 292-301 that might rather go to the discussion. If the authors prefer keeping the interpretations within the Results part, they might consider restructuring their manuscript by having a section of "Interpreted Results" and then only a "Conclusion" section (without a discussion part).

> We have decided to maintain the names of the major sections, which are standardized in this journal. We hope the interpretive aspects of the Results section help readers to make sense of the various analyses.

Discussion

The discussion is quite short and might be improved by being restructured around two or three main aspects, and further developing the implications of this research insights in each one of these. Now, the discussion lacks a broader perspective and would gain by not only stating this study work within cognitive development in general, but maybe, also, in what these insights might tell us about Indigenous learning process, by further developing on the cross cultural potential aspects, etc.

> The revised paper includes an expanded Discussion that better contextualizes the results, as well as an *Inclusion & Ethics* section in which we clarify that "The research concerns fundamental cognitive processes across human groups and so is relevant to people broadly, rather than to any specific community, including Tsimane' communities."

Add reference for the sentence finishing line 561.

> Ok.

Methods:

- Specify who conducted the data collection and provide their qualifications or roles.

> The revised paper includes an "Author contributions" section and "Acknowledgments" section that describes these roles.

- Include the age range of participants in both experiments (not just average ages).

> Done.

- Clarify the timeline of Experiment 1.

> Done.

References:

A lot of typographical errors remain in the manuscript. For instance, the authors should change their "???" by actual data. This is the case line 966, line 1018, 1027, 1032, 1086, 1101,1251, 1311, 1317, 1351, 1358, and 1360
Line 993: "theory" is repeated. Delete one

> Done.
> We thank the reviewer for these helpful corrections and suggestions!

**REVIEWER COMMENTS**

**Reviewer #2 (Remarks to the Author):**

After revisions I am a bit more confused by the author's overall theoretical outlook (particularly why they take this to be consistent, never mind supportive of Whorf) but I don't think that is a reason to hold up publication of this very interesting and accessible work. I think it is acceptable as is.

> We apologize for the confusion - fortunately, these Whorfian questions feature only in these reviews, and not in the manuscript. In short, Whorf says that cognition is shaped in part by the structure of language, a claim that is consistent with the kind of flexible, data-driven learning mechanisms we show evidence for in the paper. In any case, we thank the reviewer for the interesting discussion and positive evaluation.

**Reviewer #3 (Remarks to the Author):**

Responding to the points in order

1 - The revision addresses my concern, thank you.

> Excellent.

2 - I take the point that 'language learning' applied to a sufficiently broad set of domains becomes de facto domain general.

However the authors argument that the task was somehow non-communicative makes no sense to me: They admit that "the context of any experiment is in some way communicative" but then say "our tasks were not." I agree with the first statement, and don't see how the second can possibly follow. Nor do I see how the nature of the patterns or lack of feedback make the task non-communicative. The experimenter has asked the participant to do something, and the participant will (correctly) assume that the experimenter will check and interpret their performance in some way. I fail to see how this is a non-communicative context — or at the very least how the authors can rule out that participants interpret it as one.

> We agree that our statement was contradictory and apologize for the confusion. We have removed this wording from the revised manuscript. Our experimental context was communicative in that experimenters and participants communicated about the task. As the reviewer points out, the experimenters gave verbal instructions and made demonstrations; the participants sometimes asked clarification questions and likely looked to the experimenters for signs of approval. In this way,

experimental contexts may be communicative as long as experimenters and participants interact. However, our task did not require speaking aloud, writing, gesturing, or coordinating action with another person. Neither the patterns nor the blocks signified anything – squares were squares and circles were circles. If participants construed their patterns as somehow communicative, it is not clear what information they might be communicating with the patterns: there was no message to convey.

That said, we agree that we cannot rule out that participants performed our task using the same learning mechanisms used for natural language and other "systems of symbolic communication," which we believe is the central point of the reviewer's concern. Indeed, we suspect the same mechanisms are at play in both, as well as in learning algorithms over non-symbolic actions like parallel parking and tying shoes (our Computation & Language Lab has worked on using program learning for acquiring symbolic systems of communication – e.g. Yang & Piantadosi 2022, following on Chater & Vitanyi). In response to this comment, we have modified our discussion of the results and their implications for theories of skill-learning, including language:

*The present results clarify how human learners across disparate cultures can infer even arbitrary patterns in a novel domain without instruction, knowledge of common algorithms, or large amounts of data. To be clear, these findings do not rule out domain-specific learning mechanisms, which may guide learning in domains like spatial reasoning, social relations, and natural language [143]. However, they suggest that such mechanisms are either complemented by more domain-general mechanisms or are simply not very domain-specific. For example, in principle participants could have performed our tasks using the same learning mechanisms they used to learn language, inferring the implicit rules structuring each visual pattern just as they inferred the implicit syntactic rules structuring Tsimane', Spanish, or English. However, if these learning mechanisms apply not just to natural language but also to any meaningless pattern of shapes or sequence of actions, then they may be better characterized as mechanisms for pattern learning in general, rather than for language learning per se [144].*

I'm also confused by the asymmetry in the new text provided by the authors: They point out that domain-specific mechanisms may support performance here, but are not necessary to explain it. But is the same not true of domain-general mechanisms? Given the communicative nature of the context, and the obvious potential of patterns to act as symbolic language, can we not just as well say that performance could be supported by domain-general mechanisms, but that these are not necessary to explain it? I feel like the broader point may be that we don't have sufficient conceptual clarity on the difference between 'truly domain general' and 'broad but domain-specific' capacities. If this is the case, then it feels like the heavy emphasis specifically on domain generality should be softened (e.g. to 'broad, potentially wholly domain-general capacities')

This asymmetry derives in part from the claim that, all else being equal, theories positing many, specialized mechanisms are less parsimonious than theories positing one (or few) mechanisms. This claim is assumed in many nativist theories of cognition, which posit innate, domain-specific learning

mechanisms in part because they assume the cognitive ability in question (e.g. language, counting) is too sophisticated to learn from the available data without such dedicated mechanisms (or knowledge). This argument is weakened to the extent that children can learn arbitrary structures from tiny amounts of data in domains that lack such dedicated mechanisms, a point we clarify in the revised discussion:

*Likewise in the domain of number, scholars have long attributed children's counting abilities to innate neural mechanisms, in part because of the difficulty of the computational challenge such algorithms pose to the learner [101]. This argument is undermined by our results, which show that children can learn a variety of such algorithms from a single observation, even before they have learned to count.*

We agree that the dichotomy of "domain-specific" and "domain-general" mechanism is likely an oversimplification. In response to this comment, we have softened our claims about domain-generality, in part by construing it as a continuum, rather than a simple dichotomy. For example, in the revised Discussion, we write "However, they suggest that such mechanisms are either complemented by more domain-general mechanisms or are simply not very domain-specific" (see above).

3 - I don't see how the additional paragraph addresses the concern about formal schooling driving the observed effects. As I mention in the original review, even very limited amounts of formal schooling have been shown to produce qualitative differences in performance (see Luria's work, Cole et a., 1971; Sharp et al., 1979 etc.) The difference need not be in the learning of new representational systems (e.g. number) — it is plausible that simply learning to look for the kind of 'correct' or 'perfect match' solution within decontextualized tasks emerges as a kind of activity from participating in school.

The paragraph added by the authors does not address this concern, nor does it acknowledge the fact that the main effect seems to be confounded with schooling exposure. Instead it appears to gloss over this issue, gesturing towards the fact that a wide variery of experiences may 'sharpen program-induction abilities.' Surely this begs the question by presupposing the very program-induction abilities the authors claim to be demonstrating?

I see this as a crucial point to address in the final manuscript — even if only to say that future work will have to disentangle the present results from the effects of formal schooling. (Though of course I remain open to new arguments why this may not relevant after all).

Note, as I will mention below, I don't actually think that schooling can produce program-induction, or any other new 'fundamental' capacity. My point, rather, is that schooling has been overwhelmingly shown to impact performance on decontextualized tasks with arbitrary content, and thus should be seriously considered as a factor in the pattern of data presented here.

We agree that education could play an important role in participants' pattern induction abilities in Experiment 2. In response to this comment, we have included new plots of both accuracy and algorithmic regularity by years of schooling (and by age) in each experiment in the *Supplementary Information*, and we report the effects of both schooling and age in the main text. To summarize, we

find that performance is high even among our youngest and least-schooled participants, with some reliable effects of age and schooling on performance. Specifically, in Experiment 1 we found an effect of formal schooling on accuracy, but not when controlling for other variables like age, and no effect of schooling on algorithmic regularity, even when it was the sole predictor. In Experiment 2, we found a reliable effect of schooling on edit distance, controlling for other variables, and no effect on algorithmic regularity, even when each was the sole predictor. In addition to reporting these effects in the main text and plotting them in the Supplemental Information, we have also added to the Discussion the reviewer's point (and related references) about potential effects of schooling:

*Algorithmic abilities may also be influenced by formal and informal instruction [91, 130, 131], in which teachers and caregivers make explicit the algorithms underlying cultural skills like adding fractions, tying shoelaces, or building canoes. Although our analyses showed limited effects of formal schooling, even small amounts of schooling can have large effects on children's performance in some tasks, including generalization tasks [130, 132, 133]. Future work should more closely examine the effects of education on the type and extent of generalization in pattern-induction tasks.*

4 - An excellent clarification, thank you.

Thank you!

5 - First, a minor point on stimulus familiarity: Inhibition of relational reasoning by superficial similarity has been shown specifically when superficial matches compete with relational ones. Simply being familiar with the materials has not been shown to inhibit relational reasoning with them — as far as I am aware. Nor do I understand why it would do so (e.g. would a mathematician very familiar with numbers be less likely to treat them relationally? Or looking through a few hundred Raven's matrices stimuli inhibit performance on the test?) That being said, the authors' arguments don't seem to rest on this point, so I am happy to leave it be.

Stimulus familiarity is relevant to our study not because familiarity would inhibit relational reasoning, but because it could in principle facilitate performance in our task. For example, if we tested US American children in the algorithm of washing the dishes, their performance would likely be improved by previous experience observing parents wash dishes. We avoid such familiarity effects by testing participants using physical stimuli and abstract patterns that are unlikely to be familiar.

My more pressing, and general, concern is the following:

In no way do I wish to question Tsimane children's abilities to match abstract patterns — I have no doubt they are able to do so. My broader concern is that this point is an obvious one, given e.g. children's obvious ability to learn any number of languages and cultural patterns. In other words, what is the current study telling us that

observing the wide variability in languages and pattern-making across the world's cultures does not? Or, perhaps more specifically, the fact that children rapidly assimilate to new languages and cultures when travelling or being adopted?

After all, when faced with a wholly new culture the adopted child learns tasks which are just as novel and arbitrary as the task used here. If this was somehow impossible the relevant literatures would presumably have been full of reports and attempted solutions had this been a major barrier for their development and adaptations. Instead, my impression of such work is of consistent evidence that children rapidly and flexibly adapt to even extremely variable cultural contexts.

This is not to say the present work is not saying something new. Rather, I think the contribution lies in providing a rigorous demonstration of some of these apparent capacities. To the extent that is the case the more appropriate framing, I would argue, is something like:

"Clearly humans across the lifespan are able to learn and adapt to arbitrary patterns and tasks, as evidenced by rapid cultural learning within and and across cultural contexts. Here we provide a quantitative measure of this kind of learning, applying rigorous formal criteria to the phenomena." While this softens the impact somewhat, I happen to believe that formalizing these kinds of cross-cultural observations is very much a worthwhile goal.

> We agree that children have long proven themselves to be flexible learners, as evidenced by their ability to learn a wide variety of cultural and linguistic systems. Indeed, the paper starts with observations about the extraordinary speed and flexibility of children's learning. As we say in the Intro, "This extraordinary cognitive flexibility has allowed our species to populate a wide variety of natural environments around the world and to innovate new technological, social, and political tools...but the cognitive mechanisms that underlie this 'improvisational intelligence'...remain unresolved." So the question is not *whether* but *how* – specifically, we ask how fast and flexibly children learn and whether this learning is powered in part by inferring structured, generative processes, or by following simpler heuristics. To clarify this motivation early on, we have added to the Introduction a simple rhetorical question – "How do children learn so much, so flexibly?" – and we have reworded our Introductory summary statement, which now begins "Here, we characterized the inferential processes underlying children's skill learning by..."
>
> Although we agree that our technique provides a new way to quantify learning progress in algorithmic tasks, the results themselves also address the question above, arguably the central question for developmental theories of learning. As we say in our clarified statement of motivation: "This approach allowed us to characterize not just *whether* participants understood the sample patterns, but also *what* they learned (i.e. which logical features) and *how* they learned it (i.e. by inducing implicit algorithmic structure or tracking simple statistical regularities)." First, we show that this learning is incredibly fast in terms of the amount of data required: Children found algorithmic structure even in short patterns that are wildly underdetermined (i.e. even long sequences can reflect a wide variety of possible algorithms). Second, we show that what they learned was quite abstract, allowing them not only to imitate the pattern they saw, but to generalize its latent logical structure to new stimuli, greater lengths, and even

larger alphabets. Third, we show that it is surprisingly flexible, as learning was in a largely novel domain across a diversity of patterns with arbitrary algorithmic structure. And finally, we show evidence that this fast, flexible learning is likely powered – at least in part – by program induction, a way of learning that is about inferring hidden rules, not just imitation or association. As we say in the Discussion, “These findings suggest that human skill learning can be understood as a form of algorithm discovery: Presented with small amounts of data, children across cultures induce the abstract logical structure therein, constructing ad-hoc explanatory theories without instruction, feedback, or knowledge of other common algorithms.”

In summary, I support the publication of this piece, but strongly suggest the addition of three caveats
1) there is much work to be done teasing apart 'truly domain-general' v. 'domain-specific but broad' mechanisms, and including perhaps some conceptual clarification
2) formal schooling remains to be fully controlled for in future work
3) the general capacity for learning patterns is already evident from cultural observation, and the contribution of this work is to formalize this observation

We thank the reviewer for these thoughtful comments. To summarize, we have addressed each suggestion as follows.

1) We have reframed this distinction as a continuous one, not a simple dichotomous one. We have also expanded our discussion of how learning mechanisms for domains like language and counting might apply to other domains as well, and what this means for their domain-specificity.
2) We have added the effects of formal schooling (and age) in new results and supplementary plots, and acknowledge the possibility of robust effects of schooling in our revised discussion.
3) We have clarified how our study not only offers a novel way to quantify children’s learning, but also allows us to characterize the cognitive processes that underlie that learning.

**Reviewer #5 (Remarks to the Author):**

Dear Authors,

I am not one of the original reviewers on this paper. Given that, I am applying the same policy that I prefer applied to my own work when new reviewers are brought on at the revision stage, which is: It is not my role to introduce new problems, but to go over the original paper, the reviews already in hand, the revision, and to see whether the revision addresses the issues. Obviously, if some extreme issue was missed by previous reviewers that needs to be mentioned, but other than that I think the role is more limited.

With that in mind:

Briefly, I think that: this is a great manuscript (before the revisions and certainly after them), the revision addresses the major issues raised by previous reviewers (or adequately explains why they were not addressed); it should be accepted, and will make a valuable contribution of interest to many people in the field.

> We thank the reviewer for this fair-minded approach and positive evaluation!

====

With that in mind, I have a few small-level comments that the authors should feel free to address if they so choose, but these are all minor and do not stand in the way of acceptance/publication.

1. "[languages of thought] can be universal in their expressive power, capable of representing all possible computations — Here and elsewhere, there's very little consideration for the fact that something may be _in principle_ learnable (or computable, or representable) but that it might take eons to find the relevant program in a language that is ill suited to the task. 'Turing complete' turns out to be a very low bar, one that be achieved by the right combination of buckets and sand contraptions, and recently it was noted (jokingly) that Powerpoint is Turing-Complete. The question is often turns on whether there are relevant primitives or pre-processors of stimuli or learning mechanisms or search mechanisms that would allow the inference of a program in relevant time. I know the authors are highly aware of this fact, having worked on it themselves, and it isn't terribly important to expand on, but it may be worth remarking on the issue here and elsewhere as relevant (see another example below).

> We agree this is an interesting and important aspect of any model of cognition, including LOT models, that involves searching vast hypothesis spaces. Although a full discussion of this topic is beyond the scope of this paper, in part because we don't have data on the inference technique children use on these spaces, in response to this comment we have added this point (and several relevant papers) to the discussion:
>
> *An important question for future work is how people search large (or infinite) hypothesis spaces like those presented by our patterns and how the set of conceptual primitives in the language of thought shapes what people infer in a particular domain [13, 127–131].*

2. Related to the above, I get that the authors are not making strong claims about *which* LoT is being used by children, and it is sufficient to argue that there IS one (as opposed to other forms of learning). Still, I was highly interested as a reader who cares about this work to know a bit more which primitives are likely most relevant. Again, this raises the problem that a teeny-tiny LoT is likely able to learn all of these patterns in principle given sufficient time, but some LoTs are more naturally suited to it than others, and it is very interesting to consider

whether this work is telling us anything about what the likely primitives of that LoT are (a-la other work by Piantadosi and his colleagues on concept learning).

> We agree this is an interesting question and our lab has attempted to tackle it in some other domains, but they tend to require huge amounts of data, beyond what we have available with this group. However, since the question is important, we now include it in the discussion (see above).

3. To the paragraph staring in 210 and ending in 219 ('If program induction drives the acquisition of domain-specific algorithms like the counting algorithm...), this again seems to me like a false dilemma, as though what is possible is either 'Program Learning' or 'Association Learning' and then success or failure on a given task may show that for *this* task 'Program Learning' wasn't available. It is also possible that one's LoT has certain primitives and operations and pre-processors that make some domains much easier to learn or figure out in a given amount of time compared to others.

> To clarify, this paragraph is intended to point out that program induction cannot be assumed in these populations or tasks, and that our interest is not whether children succeed or fail but in characterizing the processes that likely produce their responses. That said, we agree that if we had found no evidence of program induction here, it would not be safe to conclude that children do not do program induction. Rather, the specific features of our tasks, populations, primitives, and testing context might have precluded the use of latent program induction abilities.
>
> In response to this comment, we have revised the paragraph in question, which now ends: "Likewise, children might be unable to apply any latent program induction capacities to our tasks if, for example, the specific stimuli we chose were not clearly distinguishable to them. In such cases, participants might be able to replicate some aspects of the target pattern, but unable to perform the kind of abstraction and generalization that characterizes cognition in many domains among human adults."

4. How much, if any of this was pre-registered (sample sizes, analyses, models, etc)? I couldn't figure that out quickly from the repo or reporting summary pdf, and it doesn't seem in the paper itself that anything was pre-registered. That isn't a requirement, but it is helpful to know these days what was agreed on ahead of time, how sample sizes were determined, what else was tried, and so on. If this was mentioned somewhere and I missed it, my apologies.

> Neither study was preregistered, in large part because of the nature of this type of fieldwork. Upon arrival in the field, we work with native Tsimane' research assistants to pilot and (re)design the tasks and data collection procedures, in an effort to make them more culturally appropriate, a process that can take days or even weeks. We also have little control over when or how long our testing takes place, as progress depends on weather, road conditions, travel-related illness, and local events. This makes

preregistering a sample size little more than guesswork. In the revised Method section, we note the lack of registration, briefly explain the reasons, and explain how sample size was determined (i.e. ad hoc, but without knowledge of the results).

5. "Likewise here, the inductive process was not all-or-nothing: children often inferred some of the features of the sample patterns, while missing others." - this is highly interesting and relates to (2), it left the reader wondering which features were inferred and which were missed, assuming there was any systematicity to it, it can inform the LoT being used (if at all).

We agree! A partial answer can be gleaned from Figure 3a, which shows participants' accuracy in reproducing various features of the patterns in Experiment 1. But a thorough comparison of the inferred features may be best left for another study, as we suggest in our comment of important future directions above.

7. To this bit in the discussion:

" Whereas some accounts attribute humans' extraordinary cognitive abilities to qualitative differences in learning mechanisms [14, 53, 62], others attribute them to quantitative differences in information processing capacity [136]. In model systems, differences in memory alone can have profound impacts on what can be learned, holding constant the amount of data and the inferential mechanisms. In this way, program induction could be fundamental to learning in humans and non-human animals alike, despite dramatic differences in their cognitive abilities."

-- I get that this was in response to a reviewer comment, and also I appreciate the connection to previous work [specifically Cantlon and Piantadosi 2024], but it left things a little bit unclear here. Is the claim here that animals CAN produce these patterns? Or that they CAN'T produce these patterns, BUT that the reason that they CAN'T is not due to a lack of LoT but rather to information bottle-necks within an LoT? It seems the authors are trying to simply not get into it whether animals can or can't do this, saying something like "weeellll, maybe they can and maybe they can't but if they can't, we can explain that as [x]". I realize this is likely outside the scope of the current paper but such a claim would've led me to suggest the authors actually model that, meaning they be more specific about using something like their own LoT but under different memory limitations and showing the point at which it breaks down. That could produce novel predictions for what non-human animals pass or fail, or what humans can be expected to learn under memory-stress conditions.

It is an empirical question whether animals can do the kind of generalizations we find in humans, whether this ability reflects program induction, and if they can't do it, why not. The reviewer is correct that for the purposes of this paper, we wish to remain agnostic on these questions, which we hope to

pursue in future studies. Rather, the goal is only to clarify that program induction may not be specific to humans even if it is the engine of many human-unique cognitive abilities.

8. "To be clear, these findings do not rule out domain-specific learning mechanisms" -- This goes back to points (1 and 2), but here and elsewhere in the discussion, I think a better perspective on 'domain specific' knowledge is not 'domain specific *learning mechanisms*' but rather on domain-specific primitives, algorithms, and representations. Imagine you had a program-induction model that had as primitives stuff like "Object" and "Near" and "Rotated" and pre-processing that takes in pixels and produces 3D object representations. Obviously such a model would have an easier time learning stuff to do with space and physics, as opposed to other patterns outside of that domain, but it isn't because it has a different *learning mechanism*, the learning mechanism might be the same: Bayesian induction over programs via sampled search. I want to emphasize I am *not* saying that that is the learning mechanism, nor am I saying there is only one learning mechanism, I am saying it seems at least possible (and more in line with previous developmental work) to distinguish domain-specific data and patterns as being about primitives than 'learning-mechanisms'.

This is an interesting point. We agree that ultimately it will be important to distinguish differences in “learning mechanisms” from differences in perceptual/conceptual primitives. Indeed, it may well be that what differs across domains is the set of available primitives, which constrain what learners can infer even given the same learning mechanism. We leave this difficult and interesting question for future work.
